# Phased genomics reveals hidden somatic mutations and provides insight into fruit development in sweet orange

**DOI:** 10.1093/hr/uhad268

**Published:** 2023-12-28

**Authors:** Nan Wang, Peng Chen, Yuanyuan Xu, Lingxia Guo, Xianxin Li, Hualin Yi, Robert M Larkin, Yongfeng Zhou, Xiuxin Deng, Qiang Xu

**Affiliations:** Institute of Horticultural Research, Hunan Academy of Agricultural Sciences, Changsha, China; National Key Laboratory for Germplasm Innovation & Utilization of Horticultural Crops, Huazhong Agricultural University, Wuhan, China; National Key Laboratory of Tropical Crop Breeding, Shenzhen Branch, Guangdong Laboratory of Lingnan Modern Agriculture, Key Laboratory of Synthetic Biology, Ministry of Agriculture and Rural Affairs, Agricultural Genomics Institute at Shenzhen, Chinese Academy of Agricultural Sciences, Shenzhen, China; Institute of Horticultural Research, Hunan Academy of Agricultural Sciences, Changsha, China; Yuelu Mountain Laboratory, Changsha, China; Institute of Horticultural Research, Hunan Academy of Agricultural Sciences, Changsha, China; Yuelu Mountain Laboratory, Changsha, China; Institute of Horticultural Research, Hunan Academy of Agricultural Sciences, Changsha, China; Yuelu Mountain Laboratory, Changsha, China; Institute of Horticultural Research, Hunan Academy of Agricultural Sciences, Changsha, China; Yuelu Mountain Laboratory, Changsha, China; National Key Laboratory for Germplasm Innovation & Utilization of Horticultural Crops, Huazhong Agricultural University, Wuhan, China; Hubei Hongshan Laboratory, Wuhan, China; National Key Laboratory for Germplasm Innovation & Utilization of Horticultural Crops, Huazhong Agricultural University, Wuhan, China; Hubei Hongshan Laboratory, Wuhan, China; National Key Laboratory of Tropical Crop Breeding, Shenzhen Branch, Guangdong Laboratory of Lingnan Modern Agriculture, Key Laboratory of Synthetic Biology, Ministry of Agriculture and Rural Affairs, Agricultural Genomics Institute at Shenzhen, Chinese Academy of Agricultural Sciences, Shenzhen, China; National Key Laboratory of Tropical Crop Breeding, Tropical Crops Genetic Resources Institute, Chinese Academy of Tropical Agricultural Sciences, Haikou, China; National Key Laboratory for Germplasm Innovation & Utilization of Horticultural Crops, Huazhong Agricultural University, Wuhan, China; Hubei Hongshan Laboratory, Wuhan, China; National Key Laboratory for Germplasm Innovation & Utilization of Horticultural Crops, Huazhong Agricultural University, Wuhan, China; Hubei Hongshan Laboratory, Wuhan, China

## Abstract

Although revisiting the discoveries and implications of genetic variations using phased genomics is critical, such efforts are still lacking. Somatic mutations represent a crucial source of genetic diversity for breeding and are especially remarkable in heterozygous perennial and asexual crops. In this study, we focused on a diploid sweet orange (*Citrus sinensis*) and constructed a haplotype-resolved genome using high fidelity (HiFi) reads, which revealed 10.6% new sequences. Based on the phased genome, we elucidate significant genetic admixtures and haplotype differences. We developed a somatic detection strategy that reveals hidden somatic mutations overlooked in a single reference genome. We generated a phased somatic variation map by combining high-depth whole-genome sequencing (WGS) data from 87 sweet orange somatic varieties. Notably, we found twice as many somatic mutations relative to a single reference genome. Using these hidden somatic mutations, we separated sweet oranges into seven major clades and provide insight into unprecedented genetic mosaicism and strong positive selection. Furthermore, these phased genomics data indicate that genomic heterozygous variations contribute to allele-specific expression during fruit development. By integrating allelic expression differences and somatic mutations, we identified a somatic mutation that induces increases in fruit size. Applications of phased genomics will lead to powerful approaches for discovering genetic variations and uncovering their effects in highly heterozygous plants. Our data provide insight into the hidden somatic mutation landscape in the sweet orange genome, which will facilitate citrus breeding.

## Introduction

Somatic mutations, such as single nucleotide polymorphisms (SNPs), insertions/deletions (InDels), and structural variations (SVs), are common and can significantly impact perennial plants and asexual crops [1–3]. Most somatic mutations create discord between homologous chromosomes and occur in a heterozygous state [4]. Somatic mutations that appear in the germline can be inherited during sexual reproduction and can be maintained in a heterozygous state during asexual propagation [5]. Compared to sexual reproduction, asexual propagation techniques are more likely to produce heteroplasmy and chimeric plants due to the accumulation of mutations over generations [6]. In addition, propagation from meristematic tissues may produce chimeras containing genetically distinct cell lineages [7]. Heteroplasmy and chimerism give rise to phenotypic variations and therefore, provide opportunities to identify clones with advantageous agronomic traits [8–10]. This phenomenon is often observed in asexually crops and facilitates the production of elite somatic varieties [11]. Therefore, somatic mutations provide significant opportunities for crop breeding. However, investigations based on the single reference genome did not produce an in-depth understanding of somatic mutations in diploid genomes [12, 13].

Somatic mutations are overlooked in highly heterozygous genomes for two possible reasons. First, a single reference-based analysis may mask unaligned regions and thus, lead to partially ignore of somatic variations [14]. Second, high heterozygosity affects the identification of ancestral and derived genotypes [15]. In addition, genome heterozygosity can significantly impact the generation of somatic mutations in diploid crops [16–18]. Mutation rates tend to increase in genomic regions that contain more heterozygous sites [3, 16, 17]. The hybridization background can also contribute to high somatic mutation rates, such as in sweet orange and peach [3, 16]. Recent advancements in long-read sequencing technology have played a pivotal role in uncovering haplotype sequences and heterozygous variations in diploid crop genomes [19, 20]. Indeed, phase-resolved assemblies have become indispensable for producing comprehensive panels for investigating the accumulation of somatic mutations in crops [21].

Somatic mutations in a diploid genetic model are remarkably consistent with the influence of allelic features [22, 23]. Typically, somatic mutations lead to heterozygous variations in individuals [24]. If these variations affect regulatory element sequences or coding regions, they may influence the function of one allele and might lead to substantial changes in allele-specific expression [25, 26]. For example, a heterozygous somatic SNP in the coding region of a *STAY-GREEN* (*SGR*) allele reduced the capacity for chlorophyll degradation and gave rise to a brown flavedo phenotype in navel orange fruit [27]. Sometimes, SVs can cause large-scale perturbations of *cis*-regulatory regions and, therefore, may change gene expression and thus, induce phenotypes [10, 28, 29]. Long terminal repeat (LTR) retrotransposons can increase the allelic expression in response to the cold and lead to the accumulation of anthocyanins in blood orange varieties [10]. In addition, DNA methylation in the promoter region of the *MdMYB1* gene plays an important role in regulating expression and affects the color of apple fruit [30]. Although somatic variations can influence the function and expression patterns of linked alleles, these characteristics have been poorly studied in the context of phase-resolved crop genomes.

Citrus is one of the most economically significant fruit crops in the world with enormous genetic diversity among the different species, which include mandarins (*Citrus reticulata*, *Citrus unishu*, *Citrus ryukyuensis*, *Citrus depressa* and *Citrus tachibana*), pummelo (*Citrus maxima*), citron (*Citrus medica*), lemon (*Citrus limon*), grapefruit (*Citrus paradisi*), and sweet orange (*Citrus sinensis*) [31]. Pummelo and mandarin diverged 6–8 million years ago [32]. Sweet orange is a hybrid species that possibly arose from an interspecific hybridization between pummelo and mandarin thousands of years ago [33]. The hybrid genetic background reshaped important agronomic traits in sweet orange, such as fruit size and flavor [34]. Because of apomixis, a type of asexual reproduction, and grafting (i.e., clonal propagation), the genetic differentiation of the sweet orange genome was frozen as it spread globally [3, 35]. Somatic breeding techniques are influential in sweet orange [36]. For example, sweet orange varieties descended from an early somatic clone originating in South China, the levels of acid content gradually decreased, which led to the present almost acidless varieties [3]. A chromosome-level phased diploid lemon genome has recently been assembled utilizing Pacific Biosciences (PacBio) high-fidelity (HiFi) reads [37]. This achievement follows the publication of a haplotype-resolved reference genome from Valencia sweet orange (*C. sinensis* cv. Valencia), which is based on continuous long read (CLR) sequencing [13]. Here, we used sweet orange as a genetic system to investigate hidden somatic mutations in highly heterozygous genomes. We assembled a high-quality phase-resolved sweet orange genome using a combination of HiFi reads, ONT long reads, and Hi-C reads. We investigated the genetic admixtures and haplotype differences based on the phased genome. Furthermore, we developed a strategy aiming to capture hidden somatic mutations. The whole-genome sequencing (WGS) data from 87 sweet orange somatic varieties were collected to generate a phased somatic variation map. By combining allele-specific expression and hidden somatic mutations, we demonstrate that somatic mutations influence fruit development in sweet orange. Our study answered four questions: (i) How many new sequences are apparent in the phased genome compared to the previous chimeric consensus reference genome, SWOv3 [3]? (ii) How can we use phased genomics to identify hidden somatic mutations? Furthermore, can identifying these somatic mutations contribute to sweet orange breeding? (iii) What is the genome-wide variation in expression among alleles in a highly heterozygous background? (iv) Are gene expression differences influenced by alleles containing somatic mutations?

## Results

### Haplotype-resolved assembly of diploid sweet orange

We *de novo* assembled a high-quality haplotype-resolved genome of Bingtang sweet orange, a popular variety with a characteristic set of somatic lineages that originated in South China. A total of 25.2 Gb of HiFi reads (80-fold coverage) were generated using the PacBio Circular Consensus Sequencing (CCS) platform. We also sequenced 29.2 Gb of nanopore ultralong sequence reads (N50 = 53.4 Kb) (Table S1, see online supplementary material). These reads were combined for the assembly of the primary contigs. Subsequently, the 100-fold coverage of Hi-C reads were used to phase the haplotype (Fig. S1, see online supplementary material). As a result, two haplotypes were assembled with a length of 320.9 Mb and 305.7 Mb, contig N50 = 20.6 Mb and N50 = 16.4 Mb, respectively (Fig. S2, see online supplementary material). A Benchmarking Universal Single-Copy Orthologs (BUSCOs) analysis provided evidence for more than 98.5% completeness for both haplotypes (Tables S2 andS3, see online supplementary material). Additionally, the transposable element (TE) sequences accounted for 46.17% and 44.77% of the two haplotypes, respectively (Table S4, see online supplementary material). Based on the genome annotation, we predicted 30 908 protein-coding genes in one haplotype (Haplotype A) and 29 913 protein-coding genes in the other haplotype (Haplotype B). We performed a collinearity analysis for the assembled haplotypes with the previously published sweet orange genome SWOv3 [3] and identified 16.8 Mb and 19.4 Mb of new sequences that account for 10.6% of the increase in genome sequence (Table S5 and Fig. S3, see online supplementary material).

**Figure 1:**
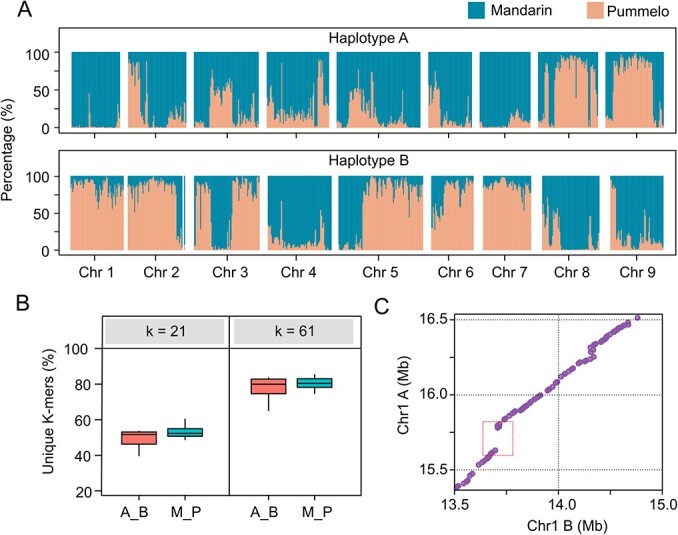
Pronounced haplotype differences in sweet orange revealed by a diploid assembly**.** (**A**) Genetic admixtures of pummelo and mandarin in the diploid sweet orange genome detected using 50-kb non-overlapping windows. The percentages from pummelo and mandarin are indicated on the y-axis and highlighted with different colors. (**B**) Comparison of unique kmers between two haplotypes (A_B) and between mandarin and pummelo (M_P). k = 21 and 61. (**C**) A 180-kb haplotype-specific region on chromosome 1 revealed by syntenic analysis.

We found that the two haplotypes are composed of different proportions of pummelo and mandarin, with mixtures occurring in different patterns among the nine chromosomes (Fig. 1A). For example, 97.4% and 14.07% of the sequences in chromosome 1 from the two haplotypes were from the mandarin genome, respectively (Table S6, see online supplementary material). We characterized the sequence divergence of the two haplotypes using unique k-mers (k = 21 and k = 61) and guide read mapping [38]. Our findings indicate significant k-mers ratios between homologous chromosomes in sweet orange that is similar to the comparison of mandarin and pummelo (Fig. 1B; Fig. S4, see online supplementary material). In addition, we collected the CHIP-seq assay data generated using the anti-*MaCENH3* protein and detected eight centromeric regions from mandarin in haplotype A and four in haplotype B (Fig. S5, see online supplementary material).

To evaluate the genome heterozygosity based on the two haplotypes, we constructed a heterozygous variation map using the whole-genome alignment and reads mapping (see ‘Materials and methods’). A total of 4.12 million SNPs and 44 731 SVs (>50 bp) were identified (Fig. S6, see online supplementary material). The whole genome distribution of SNPs and SVs were significantly correlated (R = 0.87, *P* value <2.2e-16) (Fig. S7, see online supplementary material). In addition, we identified 1723 deletions that were associated with 2722 haplotype specific genes. For example, an approximately 180-kb insertion/deletion was found to include three genes encoding proteins containing the NB-ARC domain (*HA1g13750*, *HA1g13760*, and *HA1g13780*) (Fig. 1C; Fig. S8, see online supplementary material). These large haplotype-specific sequences demonstrate the limitations of using a single reference genome to study a highly heterozygous genome, especially for detecting somatic mutations.

### Hidden somatic mutations revealed by a haplotype-based approach

We developed a haplotype-based method by integrating the two haplotypes into a complete reference genome to perform whole-genome sequence read mapping and to identify somatic mutations (see ‘Materials and methods’; Fig. S9, see online supplementary material). To evaluate the performance of the haplotype-based method, we generated simulated short reads based on this complete reference genome. Briefly, we introduced somatic mutations and simulated short read datasets at 10 different levels of coverage (ranging from 5-fold to 50-fold) to validate the power of the haplotype-based approach. We then detected somatic variations for each simulated dataset and compared results with the single reference genome-based method. We found that genotype information is important to include as part of the single reference-based mapping strategy for representing two copies of homologous alleles. The haplotype-based method focuses more on position-specific variations and therefore, detects somatic mutations by inference based on the mutations detected in sequence reads (Fig. S10, see online supplementary material). In particular, we found a clear representation without polymorphisms using haplotype-based mapping, and we found approximately 18 heterozygous SNPs per kb using single reference mapping (Fig. S11, see online supplementary material).

**Figure 2:**
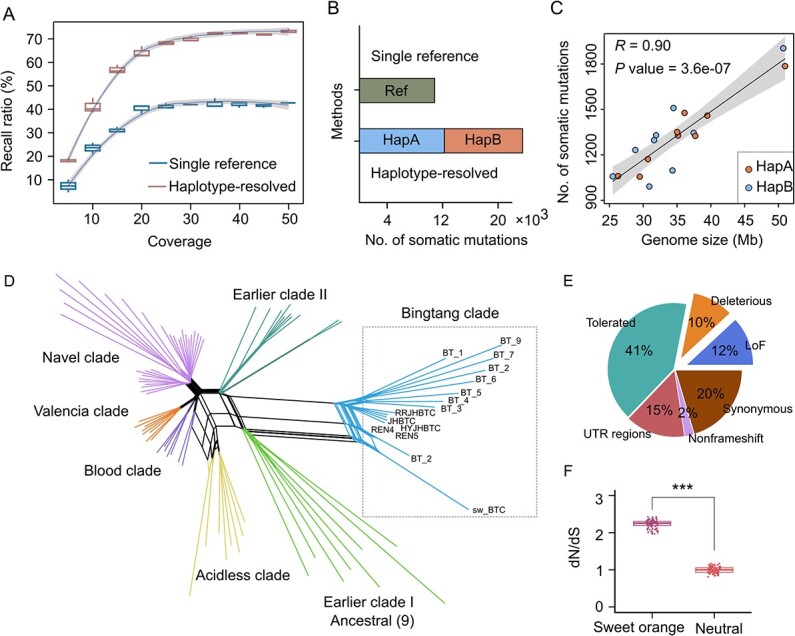
Detection and characterization of somatic mutations in sweet orange. (**A**) Evaluation of somatic mutations using a single-reference genome and haplotype-based mapping methods. The percentage of detected mutations are indicated on the y-axis. The simulated reads with different amounts of coverage are indicated on the x-axis. (**B**) Statistics for somatic variations in the sweet orange genome calculated using a single-reference genome and haplotype-based mapping methods. (**C**) Correlations between genome size and somatic variations in two haplotypes. (**D**) Network phylogeny analysis for somatic variations in the 87 accessions from the sweet orange population. (**E**) Annotation of somatic variations with high allele frequencies (>60 accessions). (**F**) Normalized dN/dS in a somatic population of sweet orange. The distribution of neutral somatic mutations was estimated in simulations.

We validated the method using simulated reads containing different variations. Our analysis showed that although SNPs and InDels were reliably detected, SVs were difficult to identify. We focused on the somatic SNPs and InDels and then performed an F-measure to calculate the recall ratio. We found that the haplotype-based method required higher coverage (~35×) to reach equilibrium but achieved a greater recall ratio (72%) compared to the single reference method (Fig. 2A). We were able to detect more somatic mutations as read coverage increased (≤35-fold) (Fig. S12, see online supplementary material). Although 75% of somatic mutations were identified using the single reference-based mapping strategy, we found that only 54% of the genotypes were correct (Fig. S13, see online supplementary material). Furthermore, we collected whole-genome short reads from 87 somatic accessions of sweet orange, including nine newly sequenced samples, with an average coverage of 45-fold (Table S7, , see online supplementary material). Using the haplotype-based mapping method, we inferred an allelic somatic mutation map, including 21 204 SNPs and 2572 InDels, which is approximately two-fold larger than the somatic mutation map produced using the single reference-based mapping method (Fig. 2B; Fig. S14, see online supplementary material). The genome length is expected to be positively related with the accumulation of somatic mutations [39]. We found similar numbers of somatic variations for both haplotypes and examined the significance of the correlation between the number of variants per-chromosome and chromosome length (*R* = 0.90, *P* value = 3.6e-07) (Fig. 2C).

Simple bifurcating trees are insufficient for modeling the genetic relationships of somatic varieties in sweet oranges due to the long-term clonal propagation and global spread. Therefore, we constructed a network phylogenetic tree to infer distinct somatic lineages using the somatic variations that we identified (Fig. 2D). We found seven major clades that are consistent with previous work indicating that acidity was under selection across different clades [3]. Interestingly, we found that clades were subdivided into groups with very large distances between internal branches. To assess the genetic mosaicism in the sweet orange genome, we estimated the individual frequency spectrum using eight accessions from Earlier Clade I as the outgroup (Fig. S15, see online supplementary material). Accessions from this clade produced fruit with the highest acid content and probably represents the oldest lineage originating from South China [3]. This analysis revealed that 47.8% of somatic variations occurred at a low frequency (<3 samples called). Furthermore, we identified 877 lineage-specific somatic mutations within the Bingtang orange clade, associated with the longest branch of the reticulated phylogenetic tree (Fig. 2D; and Table S8, see online supplementary material). We hypothesized that few reversion mutations occurred at each site and that the high frequency of somatic variations are associated with selection during breeding. Annotating somatic variations with high allele frequencies—at least 60 samples called—revealed that 51% were nonsynonymous mutations, 10% of which were deleterious and 12% were loss-of-function (LoF) alleles (Fig. 2E; Table S9, see online supplementary material). Nucleotide substitution rates may vary depending on substitution direction [40]. We introduced the *Jukes-Cantor* model for correction and calculated the normalized ratio of non-synonymous to synonymous substitutions (dN/dS) [41,42]. We found a significantly higher genome-wide dN/dS value (2.24 ± 0.11) in sweet oranges compared to the neutral simulation (Fig. 2F; Table S10, see online supplementary material). The heat shock transcription factor (Hsf) gene family is reported to participate in fruit development and maturation, in particular the accumulation of citrate content in citrus [43]. We identified a heterozygous stop-gain somatic mutation in the *HsfB4* gene that disrupts a protein domain and that is prevalent in modern cultivars, based on the mutation frequency spectrum. Expression analysis confirmed that the expression of the *HsfB4* gene is elevated during the early stages of fruit development in sweet orange (Fig. S16, , see online supplementary material).

### Differential expression of alleles during fruit development

**Figure 3:**
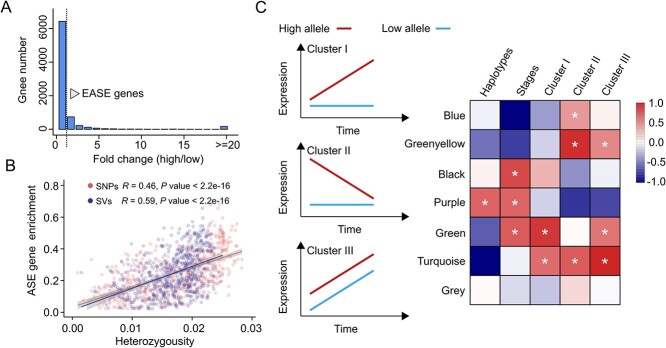
Allele-specific expression during fruit development in sweet orange. (**A**) Distribution of fold changes in expression. The fold change in expression for the allele expressed at relatively high levels compared to the allele expressed at relatively low levels is indicated on the x-axis. The threshold for extremely different fold change (EASE) genes is highlighted. (**B**) Correlation analysis between allele specific gene expression and heterozygous variations. The proportion was calculated based on the number of differentially expressed alleles and the corresponding number of genes in each 500-kb window. (**C**) Three expression patterns (clusters I, II, and III) and module-trait relationships from a WGCNA. The seven modules are indicated on the y-axis. The asterisks indicate corrected *P* values <0.05.

Phased genomics provides critical information, such as information on quantitative differences in expression of different alleles. Therefore, we constructed an allele-specific expression (ASE) dataset using expression data from sweet orange fruit at five developmental stages (90, 120, 150, 180, and 210 d after blooming) (Table S11 and Fig. S17, see online supplementary material) [44]. The heterozygous SNPs were used to build the ASE index using haplotype A as the reference genome. We confirmed allelic expression differences (FDR-adjusted *P* value <0.05) during at least one stage of fruit development for 7959 genes (24.8%) out of the 16 785 biallelic genes (i.e., genes with coding regions containing at least one heterozygous SNP) (Fig. 3A). We found a significant correlation (*P* value <2.2e-16) between the enrichment of ASE genes and heterozygous variations, and this correlation was slightly stronger for SVs than for SNPs (Fig. 3B). Furthermore, a greater than two-fold difference in expression was observed for 1418 genes (FDR-adjusted *P* value <0.001), which we refer to as extreme allele-specific expression (EASE) genes (Fig. S18, see online supplementary material). We found that 756 (53.3%) of EASE genes were associated with heterozygous SVs in the putative promoter region (Table S12, see online supplementary material). We identified that the highly expressed allele of *CCD4b* from haplotype B harbored two miniature inverted-repeat transposable element (MITE) insertions (206 bp and 158 bp) in the promoter region and that transposable elements were not inserted into the weakly expressed allele from haplotype A (Fig. S19, see online supplementary material). The 158-bp MITE was previously reported to induce increases in gene expression [45].

To understand the dynamics of allele-specific expression in fruit, we quantified the expression of EASE genes during five stages of development and found three distinct expression patterns (clusters I, II, and III) using a weighted gene co-expression network analysis (WGCNA) (Fig. 3C; Figs S20 and S21, see online supplementary material). We found increased expression for only one allele in 567 of the 1418 EASE genes (40.0%, from green and turquoise blocks; FDR-adjusted *P* value <0.05) (Table S13, see online supplementary material). At the same time, the expression patterns of the two alleles showed the opposite expression pattern, which may affect the analysis of expression differences in a single reference genome. Collectively, our analyses described a complete picture of allelic expression in the highly heterozygous sweet orange genome.

### Capturing fruit size-related somatic mutations

**Figure 4:**
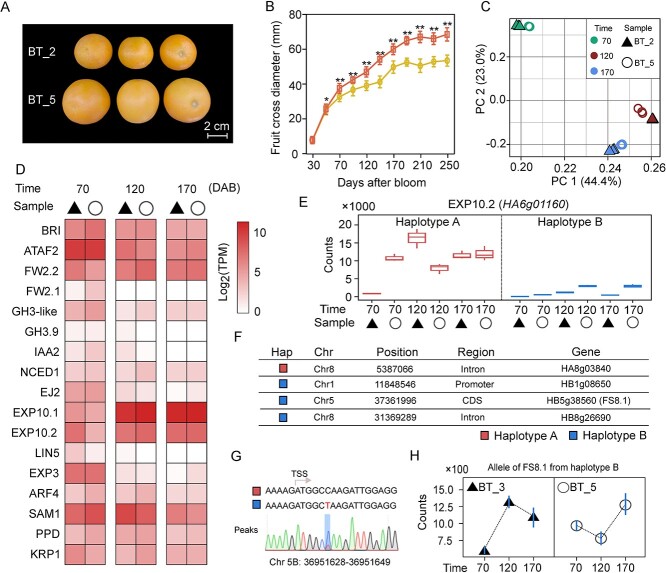
Integrative analysis of candidate genetic factors affecting fruit size in sweet orange. (**A**) Phenotypic differences between BT_2 and BT_5 fruit. Scale bar, 2 cm. (**B**) Cross sectional diameters (mm) of BT_2 and BT_5 fruit at 11 developmental stages. *P value <0.05; **P value <0.01 (Student’s *t* test). (**C**) Principal component analysis (PCA) of transcriptomes from BT_2 and BT_5 fruit at 70, 120, and 170 DAB. (**D**) Heatmap of expression levels for 17 differentially expressed fruit size and cell expansion-related genes. Genes were expressed at significantly different levels during at least one developmental period. (**E**) Expression of different *EXP10.2* (*HA6g01160*) alleles. Counts for the expressed reads from different alleles are indicated on the y-axis. (**F**) Somatic variations located in the gene region or in the 3-kb upstream and downstream regions. (**G**) Validation of somatic mutations in two alleles of *FS8.1* using Sanger sequencing. (**H**) Expression of somatic mutation related alleles from the *FS8.1* gene (haplotype B). Counts for allelic expressed reads are indicated on the y-axis.

Phased genomics is essential for discovering key variants and has implications for sweet orange breeding. We identified genes that regulate sweet orange fruit size using a set of somatic varieties with extremely tight genetic distance (Fig. S22, see online supplementary material). Fruit size differences are prominent among two somatic varieties with significant differences that are apparent at 70 d after blooming (*P* value <0.05, Student’s *t* test). The flowers and leaves are not morphologically distinct (Fig. 4A and B; Figs S23 and S24, see online supplementary material). We collected RNA-seq data from the fruit produced by both wild type (BT_2) and the large-fruit mutant (BT_5) grown in the same orchard at 70, 120, and 170 d after blooming (Table S14 and Fig. S25, see online supplementary material). Principal component analysis (PCA) indicated similar gene expression patterns in the mutant varieties and wild type during these three developmental stages (Fig. 4C). Using standard differentially expressed gene (DEG) analyses, we found that 2287, 1194, and 294 genes were differentially expressed in the fruit produced by BT_2 and BT_5 during three stages of development (FDR-adjusted *P* value <0.05) (Fig. S26, see online supplementary material). Multiple biological processes were influenced by the final fruit size and weight [46]. We annotated 113 genes that might influence fruit size and weight, including genes homologous to previous identified quantitative trait loci (QTLs) [47] that contribute to hormone signaling and the response to environmental signals (Table S15, see online supplementary material). Our results showed that 17 genes were differentially expressed during at least one developmental stage including two *FW* genes (*FW2.1* and *FW2.2*) and three *EXP* genes (*EXP10.1*, *EXP10.2*, and *EXP3*) (Fig. 4D).

Based on the phased genome, we focused on allelic somatic variations and the linked allelic expression differences. We found that 1016 (31.7%) of the DEGs were also EASE genes. In particular, *EXP10.2* (*HA6g01160*) and *FW2.2* (*HA2g13890*) exhibited relatively low expression from one haplotype, while stage-specific expression differences were determined by the highly expressed alleles (Fig. 4E; Fig. S27, see online supplementary material). If somatic mutations contribute to differences in fruit size, mutant alleles that increase fruit size should be present in the three large-fruit varieties (BT_5, BT_6, and BT_7) but absent in the other varieties. We identified 14 specific heterozygous somatic variations in three large fruit varieties. Four variations of these were specifically located in the gene body or the putative promoter region (i.e., the 3-kb region upstream of the transcription start site) (Fig. 4F; Table S16. see online supplementary material). *HB5g38560* is homologous to *AtSRG* and the *FS8.1* locus from tomato [48]. A somatic SNP was identified in the first exon of *HB5g38560* (+6 bp) in one haplotype by sequencing polymerase chain reaction (PCR) products (Fig. 4G). The allele-specific expression analysis indicated that the mutant allele of *HB5g38560* was differentially expressed at both 70 and 120 d after blooming and therefore possibly contributes to the development of the larger fruit size. This expression pattern was not captured using a standard DEG analysis (Fig. 4H). Collectively, our analyses highlight the power of using phased genomics to study the expression of somatic mutation-alleles in the sweet orange.

## Discussion

In this study, we reported a high-quality phase-resolved sweet orange genome and developed a haplotype-based mapping strategy for detecting hidden somatic mutations. The allelic features of somatic mutations and gene expression patterns were analysed together to investigate genetic factors related to sweet orange fruit size. Overall, our analyses provide a deep understanding of significant genetic variations in sweet orange and, thus, will facilitate the breeding of asexual and perennial crops.

### Applying phased genomics to discover genetic variations in crops

Haplotype-resolved genome assemblies offer a more comprehensive representation of haplotype divergence in crop genomes [49]. Recent studies have enabled phase-resolved genomes in clonally propagated crops such as kiwifruit, grapevine, and potato [20, 21, 50]. One application of these assemblies is the generation of the pangenome, which encompass sequences missing from linear reference genomes [51–53]. Here, we demonstrate the importance of studying haplotype sequences in somatic populations. We provide a detailed profile of somatic variations and allele-specific expression by discerning haplotypes. Although previous studies have examined somatic mutations based on reference genomes in peach, oak, and poplar trees [5, 6, 54], utilizing haplotype resolution for somatic mutation detection may reveal a greater number of mutations than initially anticipated.

Empirical evidence is consistent with clonally propagated crops tending to have highly heterozygous genomes [55]. Therefore, it is crucial to understand allelic signatures and somatic mutations within the context of heterozygous genomes and in particular, somatic mutation rates influenced by the genomic heterozygosity [16]. Using phased genomics to study genetic variation could reveal new insights and broadly impact the production of asexual and perennial crops. Phased genome sequencing of diverse clones will (i) lead to the discovery of somatic mutations and genes that influence important traits and (ii) help to design markers linked to causal genes for genomic selection.

### Hidden somatic mutations in sweet orange

Our haplotype-resolved genome analysis provides insight into hidden somatic variations in sweet orange. As a result of the simulations, we found that single-reference mapping can identify millions of heterozygous loci. Integrating information from both haplotypes was the first step for characterizing the mapping of mutated reads and significantly reduced the number of heterozygous loci and associated mistakes in defining genotypes. Our findings revealed that the haplotype-based mapping strategy identifies twice as many somatic mutations than were identified in the single reference genome (Fig. 2). Although the haplotype-based strategy facilitates the identification of somatic mutations, three limitations remain: (i) assemblies are incomplete in complex genomic regions; (ii) mapping errors may increase in duplicated regions, especially when the two haplotypes are integrated; and (iii) the haplotype-based method requires more read coverage.

The frequency of somatic mutation in sweet orange reveals the inheritance pattern during clonal propagation [12, 56]. Somatic mutations can accumulate in specific cell lineages as cells undergo division during development [57]. Breeders maintain genotypes using asexual reproduction methods, such as grafting, that potentially contribute to the prevalence of lineage-specific somatic mutations [54, 58]. Our findings highlight the importance of these mutations in defining the major groups of sweet oranges. Additionally, our analysis indicates that somatic mutations in the sweet orange genome were subjected to positive selection, possibly due to the intense artificial selection related to the desired agronomic traits during breeding. However, it is difficult to exclude the influence of somatic genetic drift, which can affect the accumulation of lineage-specific mutations [59].

The integration of haplotype sequences, somatic mutations and differential expression analysis of mutant alleles is a powerful approach. Due to high levels of heterozygosity, there are many EASE genes in the sweet orange genome. We found somatic mutations may influence important traits, such as fruit size, by affecting one of the alleles. Evidence for such effects comes from comparisons of allele-specific expression levels in large-mutant and wild-type fruits from sweet oranges (Fig. 4). Our analysis focused on the expression of alleles containing somatic mutations. Sometimes these differences in expression cannot be captured by a standard analysis of DEGs. Identifying the genes that affect fruit size is not straightforward because the signals that determine fruit size can act during ovary development [60]. This study did not investigate other genetic factors, such as SVs or epigenetic factors [61] but focused on a different level of regulation by accurately quantifying the relationship between somatic mutations and allele-specific expression.

### Imbalanced expression of the sweet orange genome

The heterozygous SVs might influence the regulatory elements that are critical for the expression of adjacent genes [29]. Therefore, haplotype differences in diploids have the potential to induce imbalances in the expression of different alleles of the same gene [62]. Indeed, heterozygous SVs were linked to the differential expression of two alleles [63]. For example, the insertion of a MITE element in a highly expressed allele of the *CitRWP* gene was found to induce the initiation of nucellar embryony in mandarin [28, 35]. Here, we aimed to characterize the allele-specific expression associated with SVs in the context of fruit development in sweet orange. Our findings revealed that 53.3% of differentially expressed alleles were associated with heterozygous SVs in the promoter or gene body. Furthermore, asynchronous expression patterns were observed between the two alleles of EASE genes throughout fruit development (Fig. 3). This comprehensive examination provided a holistic understanding of gene expression dynamics in sweet orange. Allele-specific regulation allows for differential gene regulation based on the specific alleles present in sweet orange during fruit development. We propose that this regulation provides alleles that confer adaptability to different environmental or genomic contexts. Sweet oranges originated from hybridization between pummelos and mandarins. In contrast to sweet orange, the different expression of alleles may not be as dramatic in pummelo. More haplotype-resolved genomes of different citrus species will provide a more complete picture of variation.

### Some genetic factors related to fruit size

Fruit size is a complex agronomic trait influenced by a multitude of genes. The hybrid genetic background of sweet orange contributes to an intricate network of interactions that influence fruit development [34]. Previous studies have indicated that signal transduction in the ovule during the early stages of development plays a crucial role in determining cell number and expansion, ultimately influencing fruit development and size at the mature stage [64, 65]. Genes involved in cell division and the cell cycle, such as *CYCD3*, *HISTONE H4*, and *WEE1*, regulate fruit size in tomatoes by controlling cell numbers [66–68]. On the other hand, candidate genes like *EXPA2*, *α-EXPANSIN*, and *AQUAPORIN*, which are involved in cell wall loosening and water uptake, determine the extent of cell expansion [69–71]. In our study, we examined a set of genes containing somatic mutations (Fig. 2). We observed differential expression of these genes related to cell number and expansion. Notably, three candidate genes involved in cell expansion (*EXP3*, *EXP10.1*, and *EXP10.2*) exhibited distinct expression patterns, particularly during an earlier developmental stage, around 70 d after blooming. Additionally, hormone biosynthesis and signaling pathways affect fruit size [72]. For instance, genes involved in the response to auxin, such as *IAA2* and *ARF4*, impact fruit size. Transcription factors are also likely to regulate multiple genes involved in development, cell division, and hormone signaling [73]. Our analysis provides evidence that the somatic mutations we studied had the greatest influence on gene expression during the early stages of fruit development (Fig. 4). Therefore, it is important to consider that key genes affecting fruit size may act at even earlier stages.

## Materials and methods

### Plant material and whole-genome sequencing

The haplotype-resolved genome was constructed from the Bingtang sweet orange lineage, namely Jinhong, cultivated at the Institute of Horticultural Research, Hunan Academy of Agricultural Sciences. Total DNA from Jinhong sweet orange was isolated from young leaves for sequencing. All plant material was immediately frozen with liquid nitrogen and ground into a powder. Subsequently, the high molecular weight genomic DNA (gDNA) was extracted following a standard protocol [74]. The concentration and quality of the stock DNA preparations were determined with a NanoDrop 1000 spectrophotometer (Thermo Scientific, USA) and using pulsed-field gel electrophoresis. The newly sequenced HiFi reads from Jinhong sweet orange were generated using the circular consensus sequencing (CCS) strategy and the Pacific Biosciences Sequel II platform. At the same time, the gDNA was used to construct Oxford Nanopore sequencing 50-kb libraries and sequenced using a GridION platform. The genomic chromosome status and associated DNA fragments were captured to construct Hi-C libraries using the restriction enzyme *MboI*, following a standard Hi-C library preparation protocol. Approximately 47.9 Gb of Hi-C reads were generated using the Illumina NovaSeq 6000 platform.

We newly sequenced nine sweet orange somatic accessions including eight from the Bingtang clade and one from the Navel clade (BT_8). There are three accessions (BT_5, BT_6, and BT_7) that produced fruit with larger sizes from the Bingtang clade. The gDNA from nine samples were collected and subjected to whole genome sequencing with an average of 35-fold coverage using the Illumina NovaSeq 6000 platform, respectively. Additionally, short reads from the previously published 78 somatic mutant accessions with more than 35-fold coverage were collected [3]. These reads were combined to construct the somatic variation map.

We collected RNA-seq data from Newhall navel orange fruit, a somatic lineage of Jinhong sweet orange, at five developmental stages (90, 120, 150, 180, and 210 d after blooming) with three biological replicates [44]. These reads were used for investigating allele specific expression during fruit development.

To characterize gene expression in somatic accessions that produce fruit with different sizes, we generated new RNA-seq libraries from the fruit of an accession that produces fruit with a normal size (BT_2) at three representative stages (70, 120, and 170 d after blooming) and fruit from an accession (BT_5) that produces large fruit at three representative stages (70, 120, and 170 d after blooming). Each data point was represented with three biological replicates.

### Genome assembly and annotation

The HiFi reads and Hi-C reads were combined to generate the primary haplotype-assembly using the Hifiasm program (v0.16) [75] with default parameters. The separated haplotypes were generated and stored in FASTA format. Subsequently, the haplotype specific kmers were inferred using the Meryl program (v1.4) [76] (https://github.com/marbl/meryl). These haplotype specific kmers, HiFi reads, and Nanopore reads were integrated to fill gaps and to generate the final haplotypes using the Verkko program (v1.3.1) [77]. The assemblies from two haplotypes were scaffolded and ordered using the RagTag (v2.1.0) [78] program based on the SWOv3 reference genome [3] (http://citrus.hzau.edu.cn/download.php). Furthermore, each haplotype was corrected with a hic contact map using the 3D de novo assembly (3D-DNA) pipeline (v201008) [79]. The Hi-C format file was visualized using the Juicebox program (v2.16.00) [80] and misaligned contigs were manually curated. Finally, the BUSCOs program (v5.4.4) [81] was used to evaluate the assembly quality.

We annotated the gene structures from haplotypes based on expression evidence and *ab initio* predictions. The RNA-seq data were mapped to the two haplotypes using the STAR program (v2.7.10) [82]. Next, the gene structure models were trained based on expression read alignments using Augustus (v3.5.0) [83] and SNAP (https://github.com/KorfLab/SNAP). Finally, homologous proteins were utilized to generate gene structures for each haplotype using two rounds of Maker (v3.01.03) [84]. To validate the gene annotation, we used the BUSCOs program to test the gene dataset from each haplotype. The TEs were identified using EDTA (v2.0.0) [85] with default parameters for each haplotype. In addition, we identified tandemly repeated sequences using the tandem repeats finder (TRF) program (v4.09.1) [86] with parameters ‘2 6 6 80 10 50 2000 -h’ and investigated the prominently repeated units for each chromosome in both haplotypes. The centromere sequences were also examined using the CENH3-based Chip-seq assay data from the mandarin Nadorcott genome [87]. The CENH3 sequences were collected from BankIt ID 2305947. These reads (including the input library as a control) were aligned to the two assembled haplotypes using Bowtie2 (v2.5.1) [38] with default parameters. MACS2 (v2.2.7.1) [88] with the additional parameters ‘-f BAM -ghs -B -q 0.01’ was used to perform peak calling.

Furthermore, we calculated the density of repeat elements including TEs and tandem repeats based on 500-kb non-overlapping windows. The number of heterozygous SNPs, InDels and SVs were also calculated based on 500-kb non-overlapping windows. To characterize the homologous chromosomes, we prepared alignments with two haplotypes using Minimap2 [89] (v2.26) and analysed the blocks of collinearity. All these data were imported to the Circos [90] program (v0.69–6) to generate a circular plot.

### Genome collinearity analysis

We analysed the collinearity of our assemblies (haplotype A and haplotype B) and the SWOv3 genome. The sequences were aligned using Minimap2 (v2.26) [89] and subsequently, structural rearrangements were found using the Syri program (v1.6.3) [91]. The collinearity analyses were plotted using Plotsr (v1.0.0) [92] and were associated with a VCF file that included candidate genomic arrangements. The BAM file was checked in the Integrative Genomics Viewer (IGV) program.

### Genetic source and divergence identification

To identify the genetic source from mandarin and pummelo, we collected *Citrus grandis* (L.) Osbeck.cv. ‘Cupi Majiayou’ v1.0 and *C. reticulata* v1.0 genomes from the Citrus Pan-genome to Breeding Database (http://citrus.hzau.edu.cn/download.php). We built a species-specific kmers dataset using the ‘count compress’ and ‘difference’ functions from the Meryl program. The sequences from haplotype A and B were split into 500-kb regions and compared with the species-specific kmers dataset. Finally, the number of kmers from each haplotype were characterized and plotted in ggplot2 [93] from the R package [94].

To estimate the mapping quality and kmers difference, we compared the kmers mapping ratio between two haplotypes (compared between homologous chromosome) and species (species-level divergence) using specific kmers with k = 21 and k = 61, respectively. The unique kmers mapping ratios for each chromosome were estimated with the number of non-overlapping kmers and the number of unique kmers.

### Construction of genomic variation maps

We used haplotype A as the reference for the reads mapping analysis. The HiFi reads were collected to identify heterozygous SNPs and short InDels using the haplotype-aware variant calling pipeline PEPPER-Margin-DeepVariant (https://github.com/kishwarshafin/pepper) with the default parameters. The heterozygous variations with high quality (tag with PASS) were kept to build a heterozygous short variation dataset. The HiFi reads were mapped to haplotype A and the SV variation maps were generated using CuteSV [95] in the HiFi reads mode. Within the heterozygous SVs, we defined large SVs (>50 bp) and the large insertions and deletions (>100 bp). The downstream statistic of SV-related genes was generated based on the insertion/deletion dataset.

### Transcriptomic analysis

The RNA-seq data from five developmental stages of sweet orange fruit were collected and mapped to haplotype A using STAR. The transcript per million (TPM) value and normalized count were calculated using edgeR (v3.42.2) [96] and Rsubread (v2.14.0) [97]. We normalized the expression matrix and performed statistics to select DEGs (FDR <0.05). To capture the high-quality DEGs, we set the expression threshold line of DEGs at TPM >1. To investigate allele-specific expression in sweet orange during fruit development, we further calculated the number of heterozygous SNPs in the coding region of each gene. The 16 785 biallelic genes were identified using the Genespace program (v.1.2.3) [98]. Based on heterozygous SNPs, we estimated the expression levels of alleles using the aScan program (https://github.com/Federico77z/aScan/). The allelic reads were identified using the heterozygous SNP dataset. Multiple testing was performed using FDR. To validate the correlation between heterozygosity (indicated by SNPs and SVs) and ASE genes, we split the genome into 500-kb windows and calculated the proportion of ASE genes in each window. The heterozygosity was estimated using the SNPs and SVs in each window. The fold-change in the expression levels of ASE genes was estimated using the average fold-change derived from ASE genes in each window. The potentially highly expressed alleles were characterized with more expressed reads and for these alleles. Fold-changes in expression were calculated based on the reads count. Genes with a fold-change >2 and reads count >100 for the highly expressed allele were defined as extreme allele specific expression (EASE) genes. We used the heterozygous SNPs to infer the relationship between the highly expressed alleles and the haplotypes and examined RNA-seq read mapping in IGV. Three allelic patterns were identified for EASE genes. We evaluated the relative significance of EASE genes and their module memberships using a weighted correlation network analysis (WGCNA) in the R package [99].

### Somatic variation detection strategy

Because sweet orange originated as an admixture of pummelo and mandarin with high levels of heterozygosity, the haplotype divergence and kmers based mapping ratios were similar to the mandarin-pummelo species level according to the sequence analysis. We were cautious about developing a haplotype-based mapping strategy to detect the somatic variations in the sweet orange genome. At the beginning, we detected short somatic variations using a single-reference mapping strategy and generated a somatic variation map with a strict filtering method. The average number of detected somatic variations for each accession was calculated to generate the base frequency. Therefore, we constructed a matrix of base mutations using the Jukes-Cantor model. To eliminate false positive mutations, we simulated reads based on sweet orange haplotypes without mutations. The falsely mutated positions that were detected were marked and removed in the downstream analysis. Subsequently, we performed 100 simulations based on the base mutation matrix and assigned the number of SNPs and short InDels to validate the detection of somatic variations using two haplotype sequences. These reads were mapped to the two haplotype sequences using BWA MEM (v0.7.17) [100]. Variation was called using Deepvariant (v1.0.0) [101]. The SNPs and short InDels were filtered with a quality <50 and a read depth <2. The recall ratio equals the number of detected variations compared to the number of assigned variations. Our simulations revealed that a haplotype-based mapping strategy could detect the mutations associated with the correct position and distinguish haplotypes. Compared with single-reference mapping, the genotype is important for identifying somatic variations. Incorrect genotypes may be filtered out with single refence mapping. The genotype was not considered during the mapping of the two haplotypes. In addition, we checked the flanking 150-bp sequence obtained for each mutation. If the sequence on the corresponding homologous region is completely consistent, it will be considered as a duplicate call, and only one mutation will be kept. Thus, more realistic mutation location information can be captured.

To test whether there is a correlation between the reads coverage and the variation recalling ratio, we simulated sequencing reads with different coverage. The coverage was inferred based on the size of one haplotype. We calculated the recal ratio using three assumptions: (i) single-reference mapping for detecting mutations with the precise genotype; (ii) single-reference mapping for detecting mutation positions not considering the genotype; and (iii) haplotype-based mapping for detecting mutations. The high depth of coverage contributes to the equilibrium of three assumptions.

### Somatic variation map in the sweet orange genome

We combined nine new sequences and previously published sequences from 78 somatic sweet oranges [3] to construct a short somatic variation map using a haplotype-based mapping method. To obtain a reliable somatic variation map for real data in practice, we additionally tested the distribution of genome-wide somatic variations. Non-overlapping 50-kb windows that were significantly out of the binomial distribution were removed. Therefore, we obtained a reliable somatic variation map for sweet orange. Furthermore, we built a network phylogenetic tree using the SplitTree program (v5.3.0) [102] based on the somatic variation map. The seven clades were inferred based on the topology.

To describe the ancestral status of variations in the sweet orange somatic population, we calculated the derived somatic mutations based on the ancestral sequences inferred from earlier clade I, which was reported as the oldest lineage from the clonal propagation of sweet orange. The individual allele frequency spectrum of somatic mutations was constructed based on 79 sweet orange accessions. To investigate the number of deleterious somatic mutations and LoF variants, we predicted the potential influence of somatic variations using the SIFT 4G algorithm [103]. First, we created genomic databases with SIFT predictions and annotated variants using the SIFT 4G Annotator. Based on the SIFT annotation database, SNPs located in CDS regions were annotated as synonymous or nonsynonymous. Second, an amino acid substitution was predicted to be deleterious if the score was ≤0.05 and tolerated if the score was >0.05. Because the sweet orange somatic population is relatively young, it is difficult to evaluate selection by directly calculating dN/dS. To evaluate the selection of genome-wide variations, we introduced the normalized dN/dS value as recommended by Martincorena *et al.* [42]. We simulated genome-wide mutations with natural effects and inferred the dN/dS value, which was the distribution of natural somatic sites. The dN/dS values were estimated based on the matrix of base mutations in sweet orange using the Jukes-Cantor model correction and subsequently, normalized to the simulated natural distribution. Given that population size influenced the pattern of somatic mutations in long-term propagated sweet orange, we cannot exclude the effects of somatic drift. Finally, Sanger sequencing was used to check the somatic mutations.

## Supplementary Material

Web_Material_uhad268

## Data Availability

Data supporting the findings of this work are available within the paper and its supplementary information files. Whole genome sequencing and RNA-seq data are accessible through NCBI under the BioProject ID PRJNA967756. Genome sequences, gene annotations, and somatic mutation maps were uploaded to https://zenodo.org/record/8016647.
