# Supplementary material for: Phased genomics reveals hidden somatic mutations and provides insight into fruit development in sweet orange
Source: Hortic Res. 2023 Dec 28;11(2):uhad268. doi: 10.1093/hr/uhad268 (PMC10873711; doi:10.1093/hr/uhad268)
Supplement: Web_Material_uhad268 [file web_material_uhad268.zip › Supplementary_figures.zip!/Supplementary_figures.pdf]

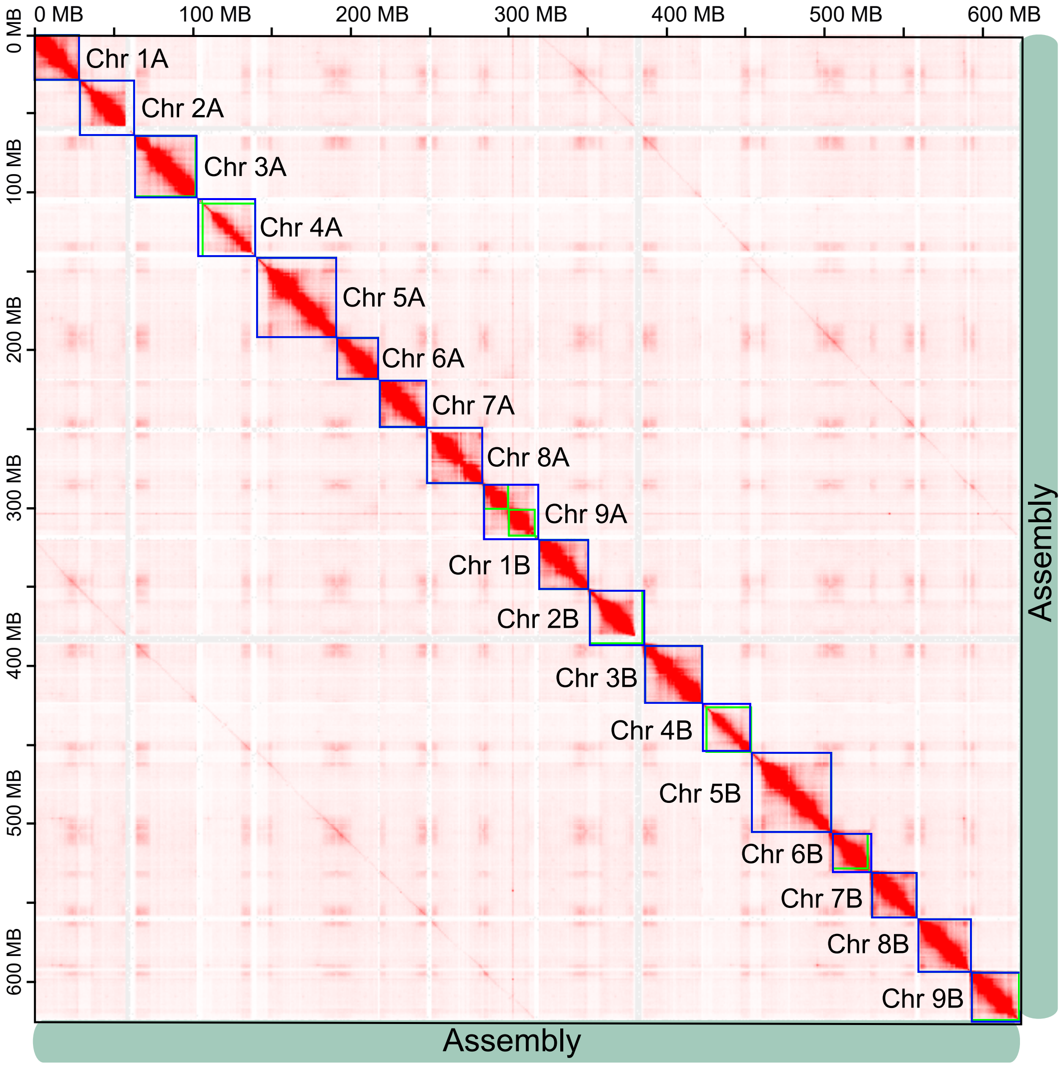

Supplementary Fig. 1. Interaction map for Hi-C reads from two haplotypes. Chromosomes from haplotypes A and B are labeled A and B.

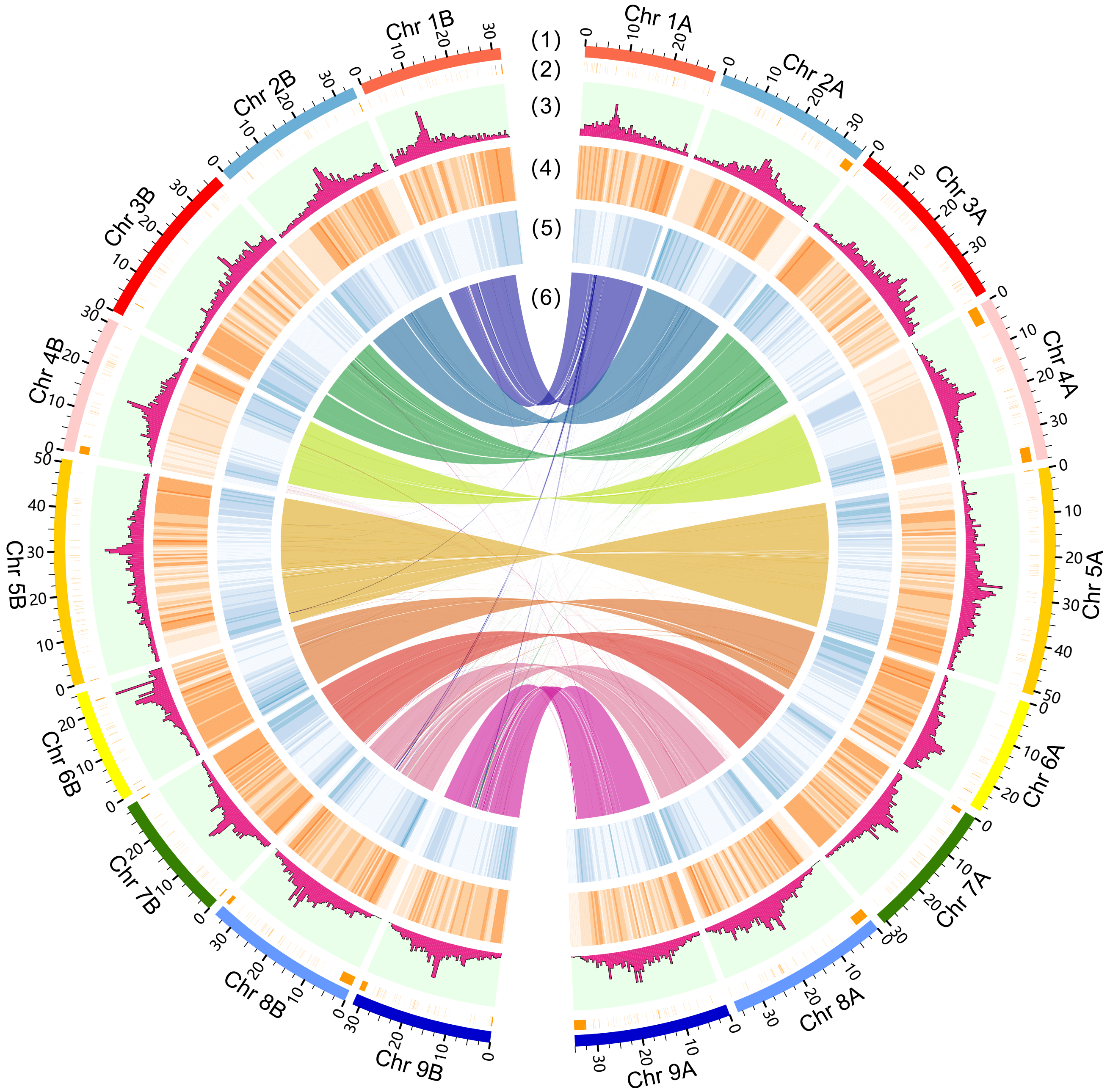

Supplementary Fig. 2. Genome assembly of diploid sweet orange (1) Circular representation of the pseudochromosomes in the two haplotypes, (2) distribution of repeat units in each chromosome, (3) distribution of repeat sequences with densities calculated in 500-kb windows, (4, 5) heatmap of heterozygous short variations (SNPs and InDels) (4), structural variations (SVs) (5). (6) collinearity blocks showing the genomic regions shared between homologous chromosomes. The band width is proportional to the syntenic block size. Statistics are based on 500-kb non-overlapping windows.

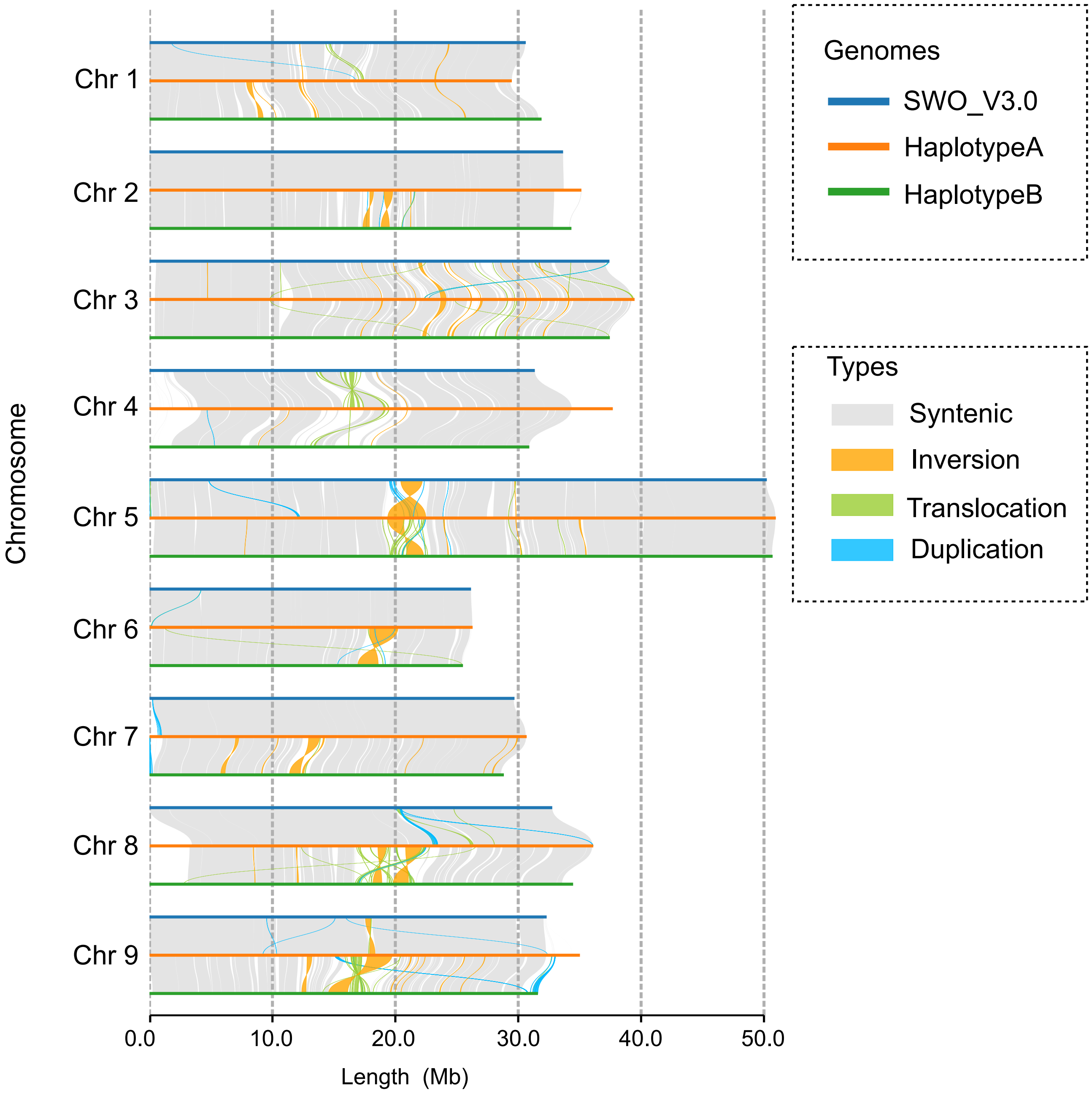

Supplementary Fig. 3. Collinearity analysis of assembled haplotypes with the previously published sweet orange SWOv3 reference genome. The analysis was performed using the Plotsr program. The structural variations (SVs) are highlighted, including inversions, translocations and duplications.

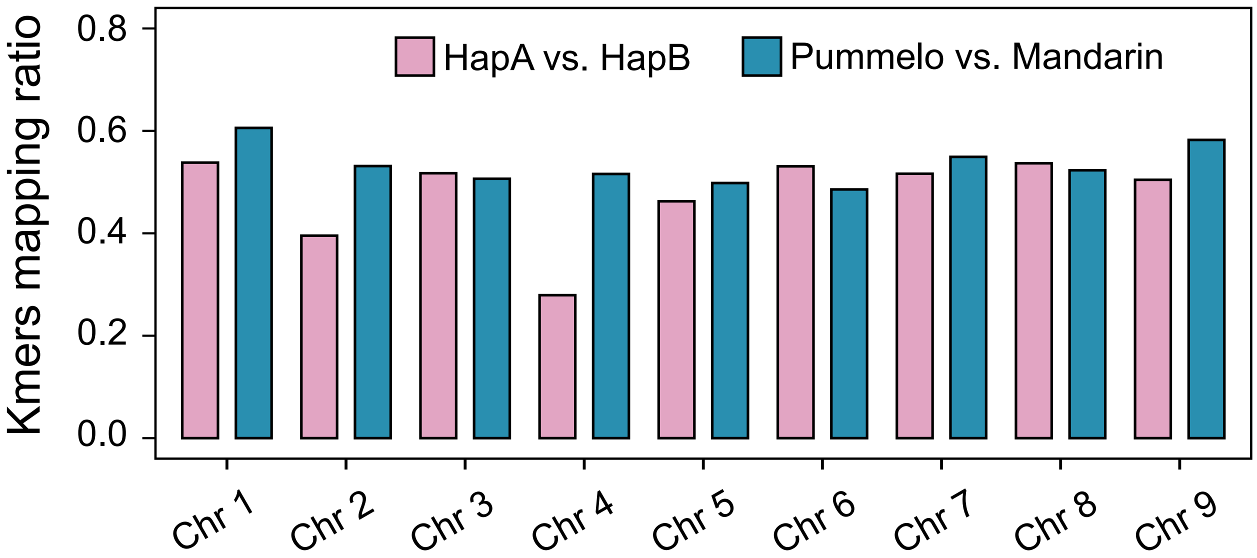

Supplementary Fig. 4. Unique kmers compared between two haplotypes and between mandarin and pummelo. The k-mers ratios (k = 21) in nine chromosomes are shown.

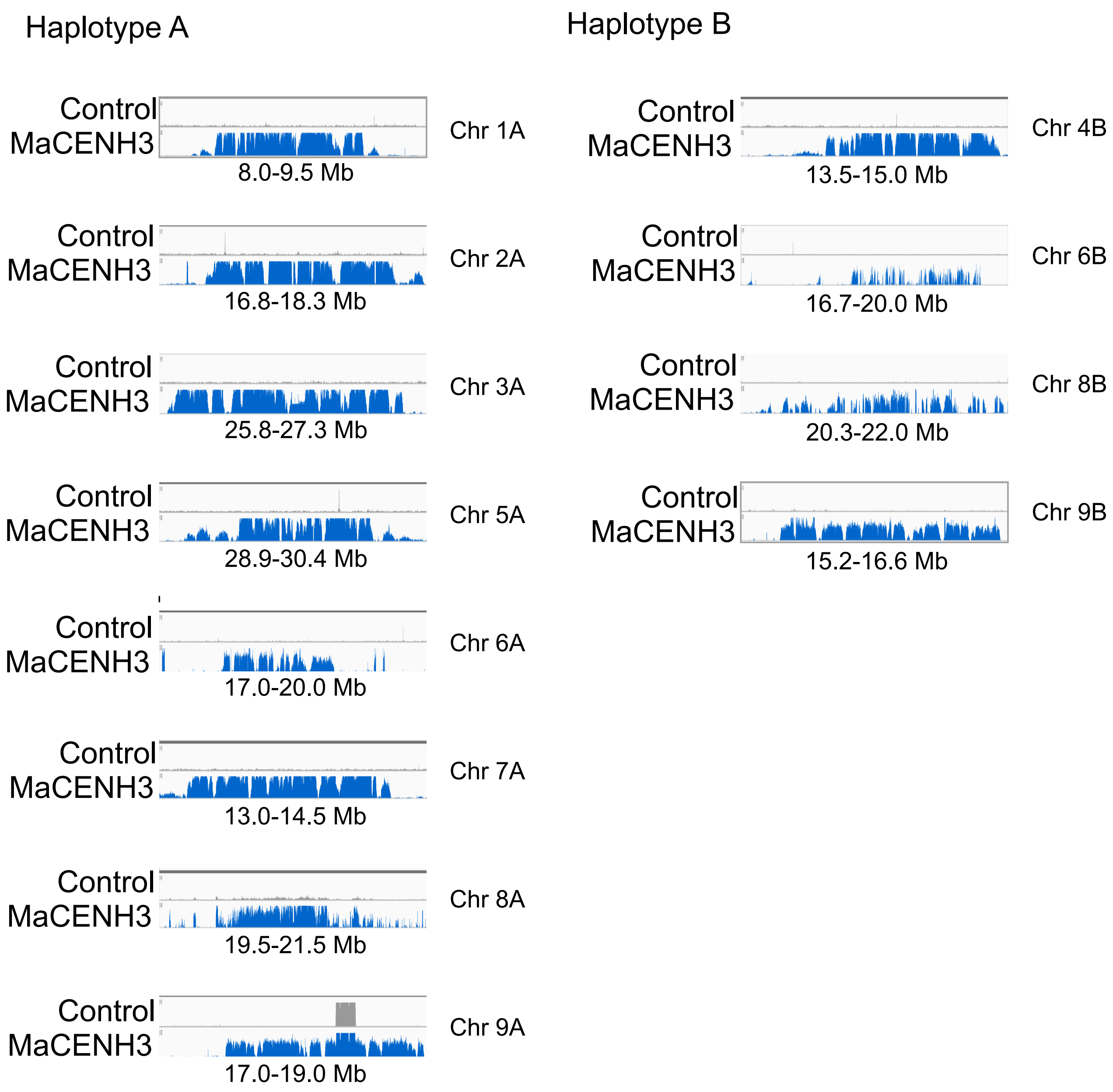

Supplementary Fig. 5. Genomic peaks from *MaCENH3*-based CHIP-seq assay data. Eight mandarin centromeric regions from haplotype A and four mandarin centromeric regions from haplotype B were characterized.

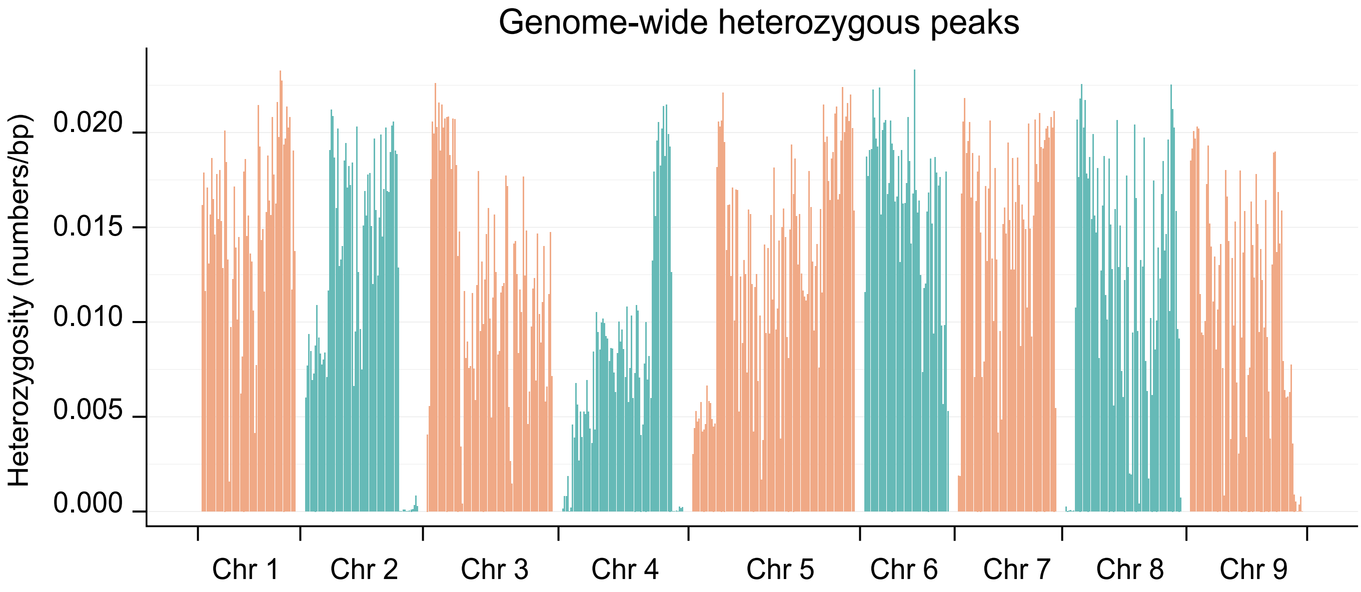

Supplementary Fig. 6. The heterozygous peaks of sweet orange genome. The heterozygous peaks were calculated based on 500-kb non-overlapping windows and the number of heterozygous variations.

Correlation analysis of heterozygous SNPs and SVs

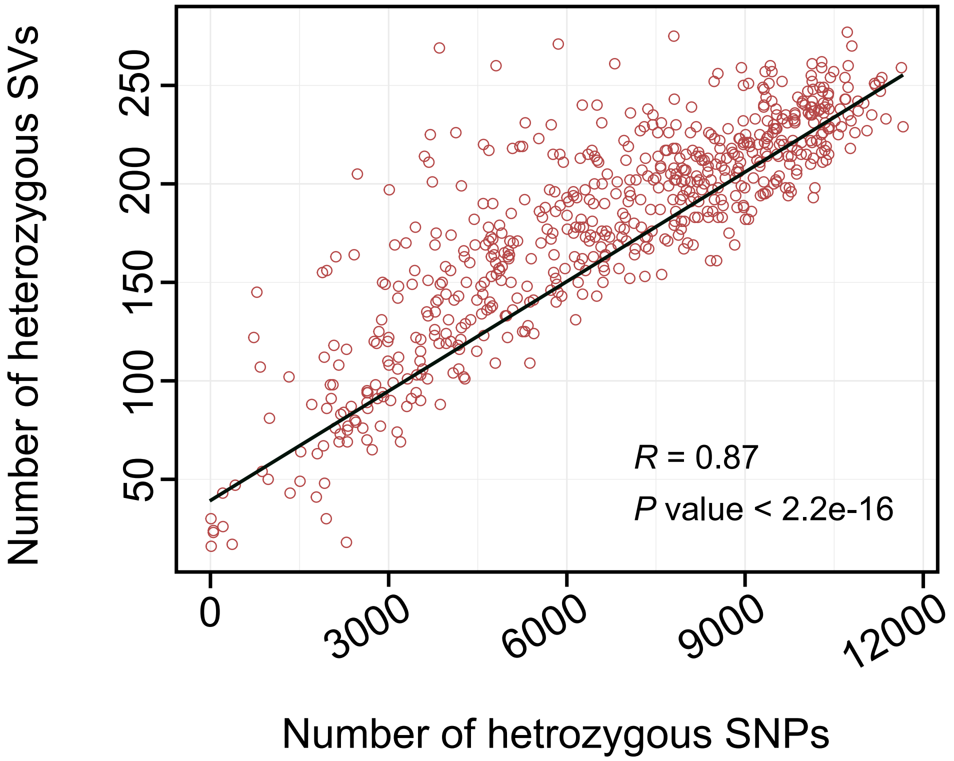

Supplementary Fig. 7. Correlations between genome-wide heterozygous SNPs and SVs. The number of heterozygous SVs and heterozygous SNPs were calculated based on 500-kb non-overlapping windows. The correlation was performed based on *Pearson's* correlation coefficient.

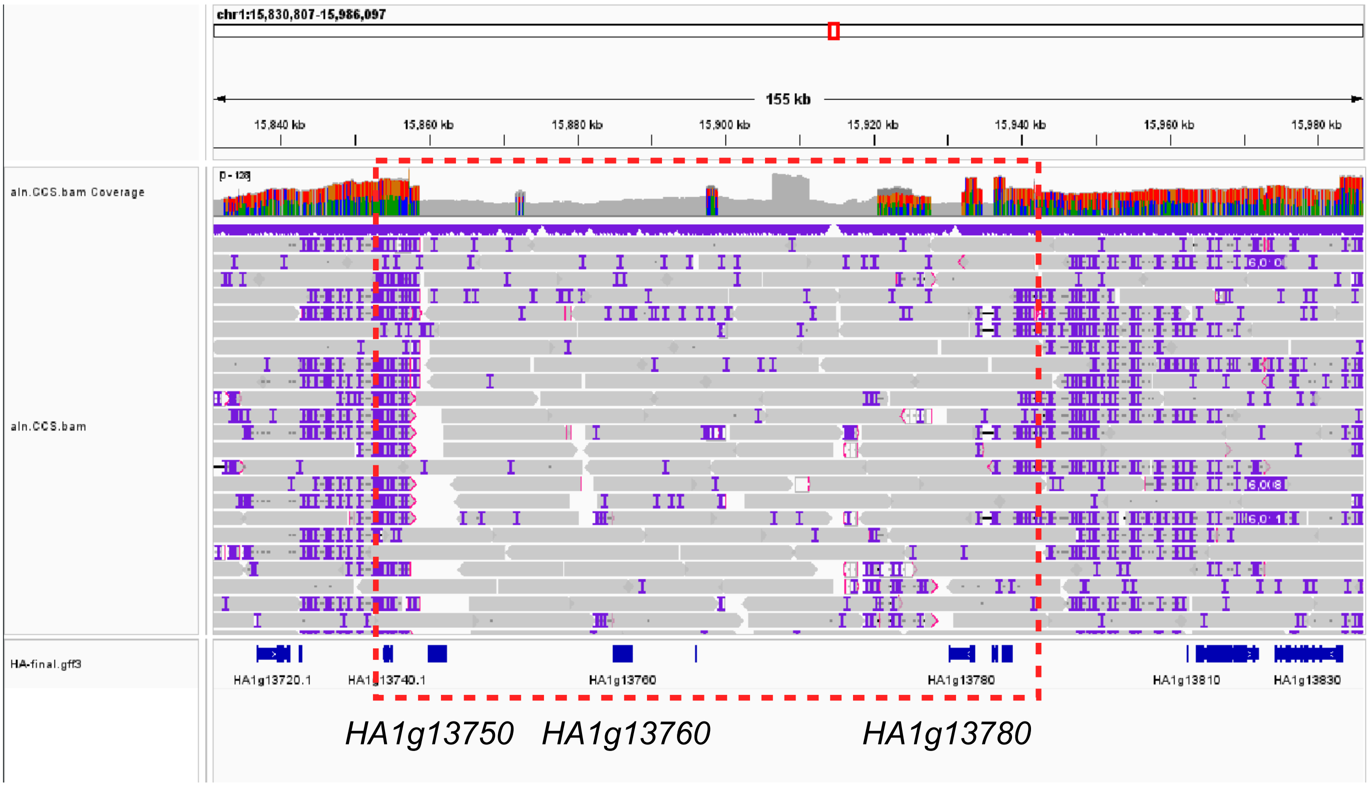

Supplementary Fig. 8. IGV plot for a 180-kb haplotype-specific region. Three genes encoding NB-ARC domain containing proteins (*HA1g13750*, *HA1g13760* and *HA1g13780*) are highlighted.

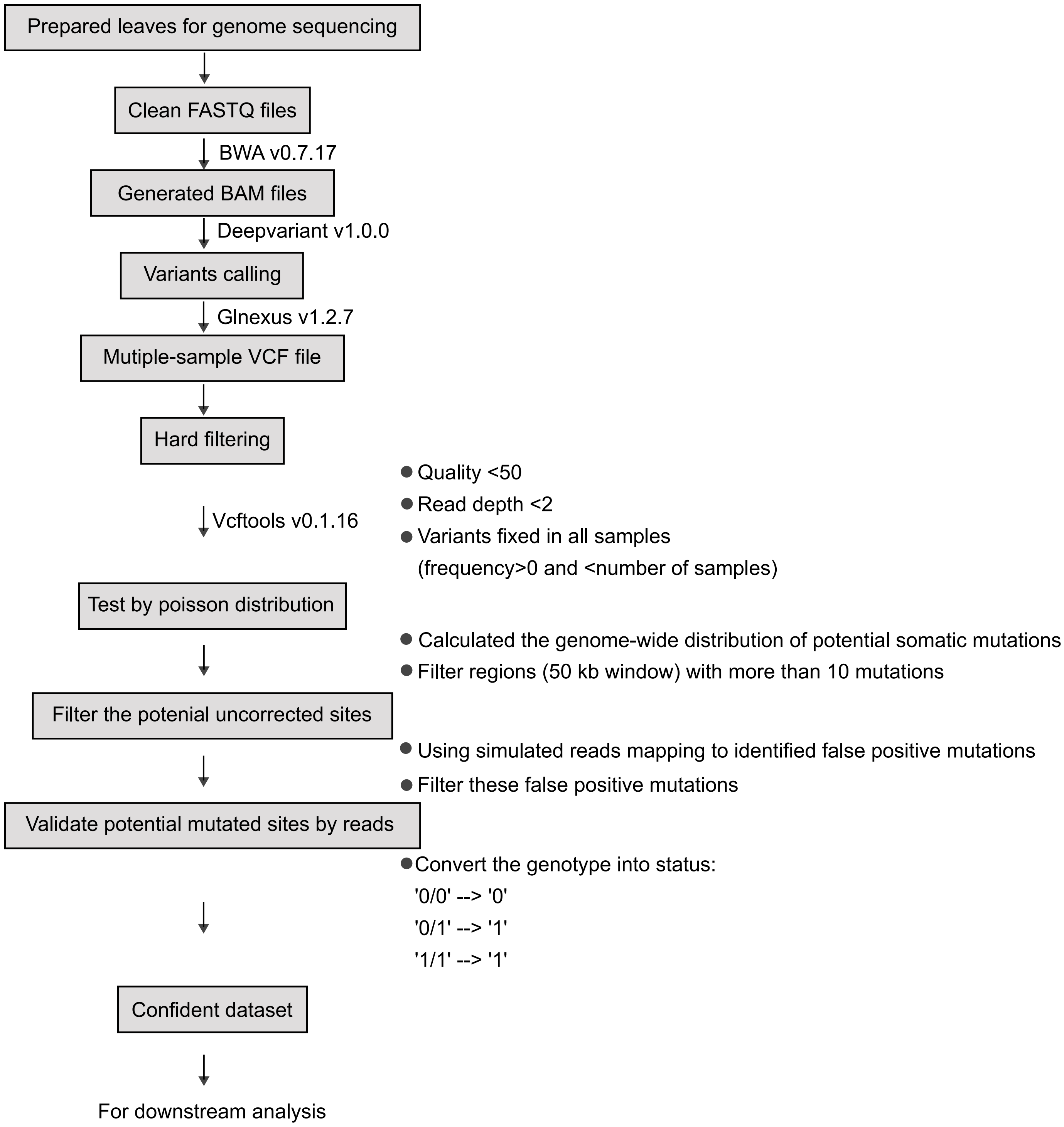

Supplementary Fig. 9. Flowchart of the pipeline for identifying somatic mutations in the sweet orange genome. This pipeline was used to construct the final haplotype-based somatic variation map.

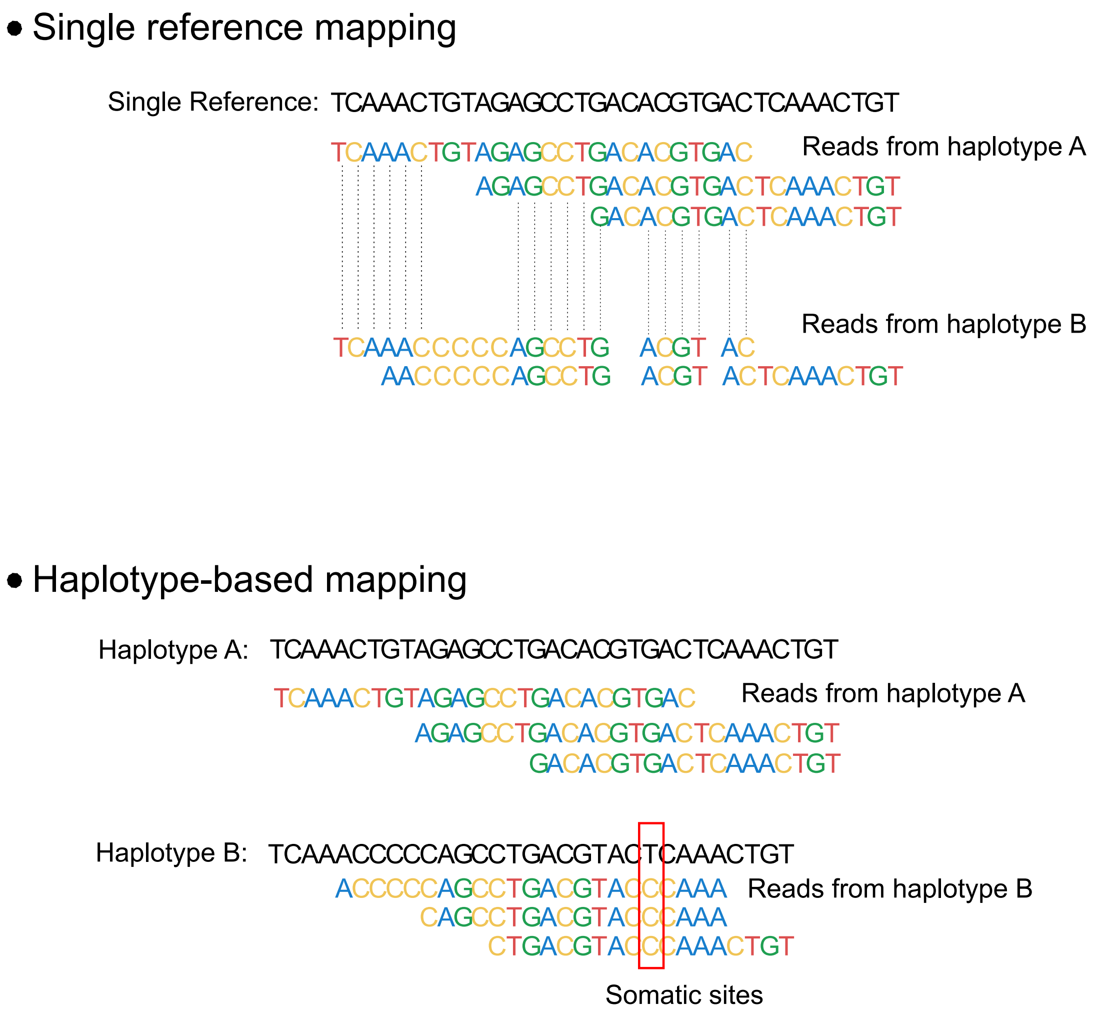

Supplementary Fig. 10. Diagram for mapping in a single reference genome and haplotypes. The target somatic mutation is highlighted using the haplotype-based approach. High-quality somatic mutations are supported at least two mutated reads.

IGV plot mapping using simultaed short reads

Haplotype A Chr1:15,776,734-15,778,002

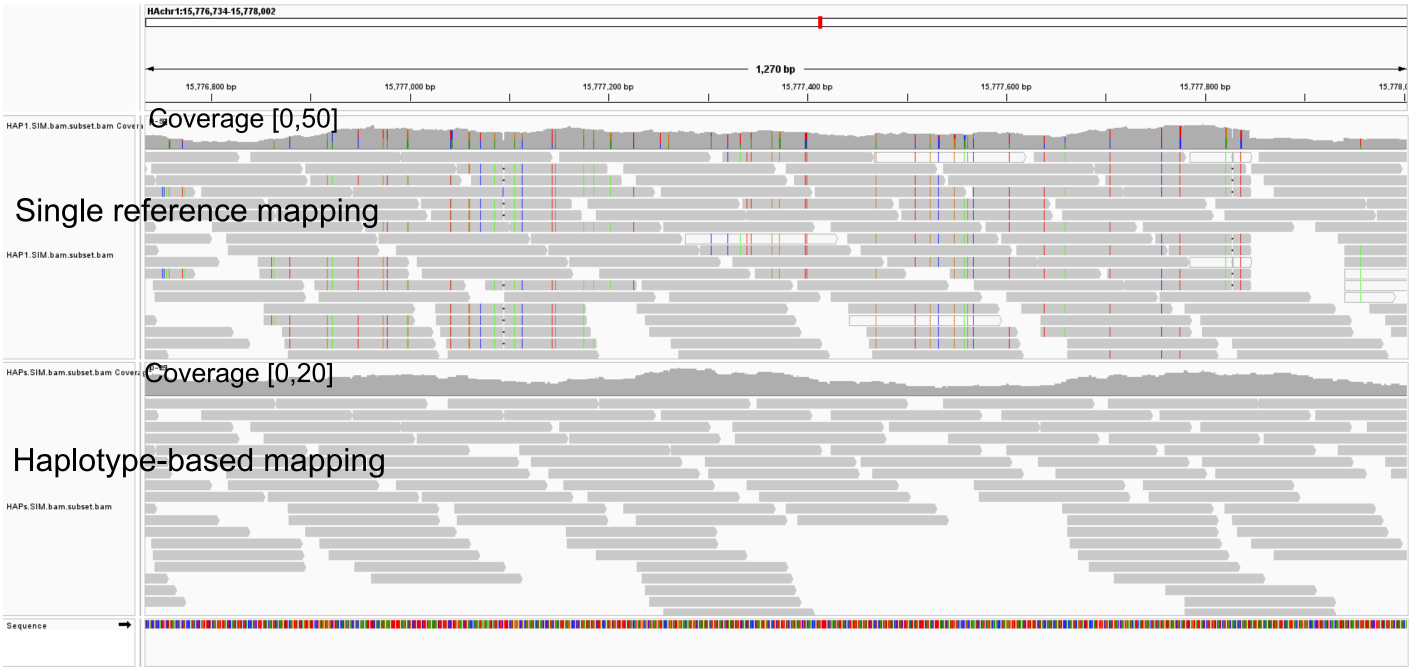

Supplementary Fig. 11. IGV plot supported the mapping results using single-reference mapping and haplotype-based mapping. The BAM files were generated based on simulated short reads using the BWA MEM algorithm. There is a clear region lacking polymorphisms in Chr1:15,776,734 - 15,778,002 bp, based on the haplotype-based mapping. Otherwise approximately 18 heterozygous SNPs with single reference mapping.

Chromosome 5 (Haplotype A)

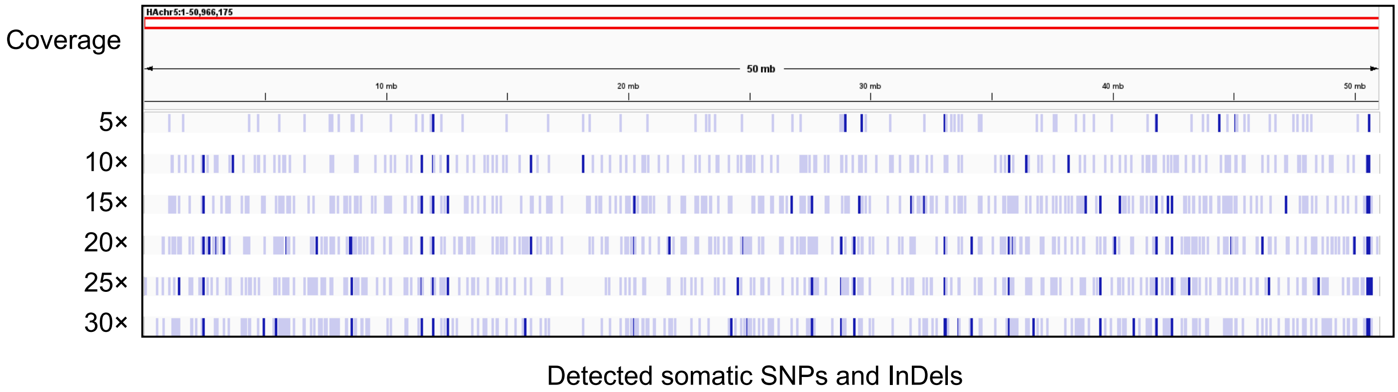

Supplementary Fig. 12. Density of somatic mutations in chromosome 5 from haplotype A obtained with different amounts of coverage of simulated reads. The results of six simulated reads at different levels of coverage were plotted using the IGV browser and the haplotype-based method.

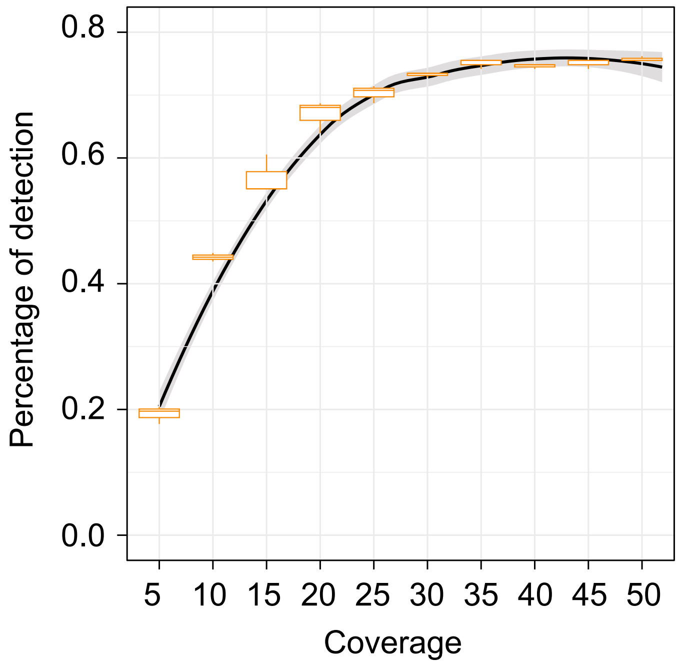

Supplementary Fig. 13. Estimation of somatic mutation locations from single-reference mapping. The percentage of detection only considers the location information of somatic mutations and is calculated based on simulated reads.

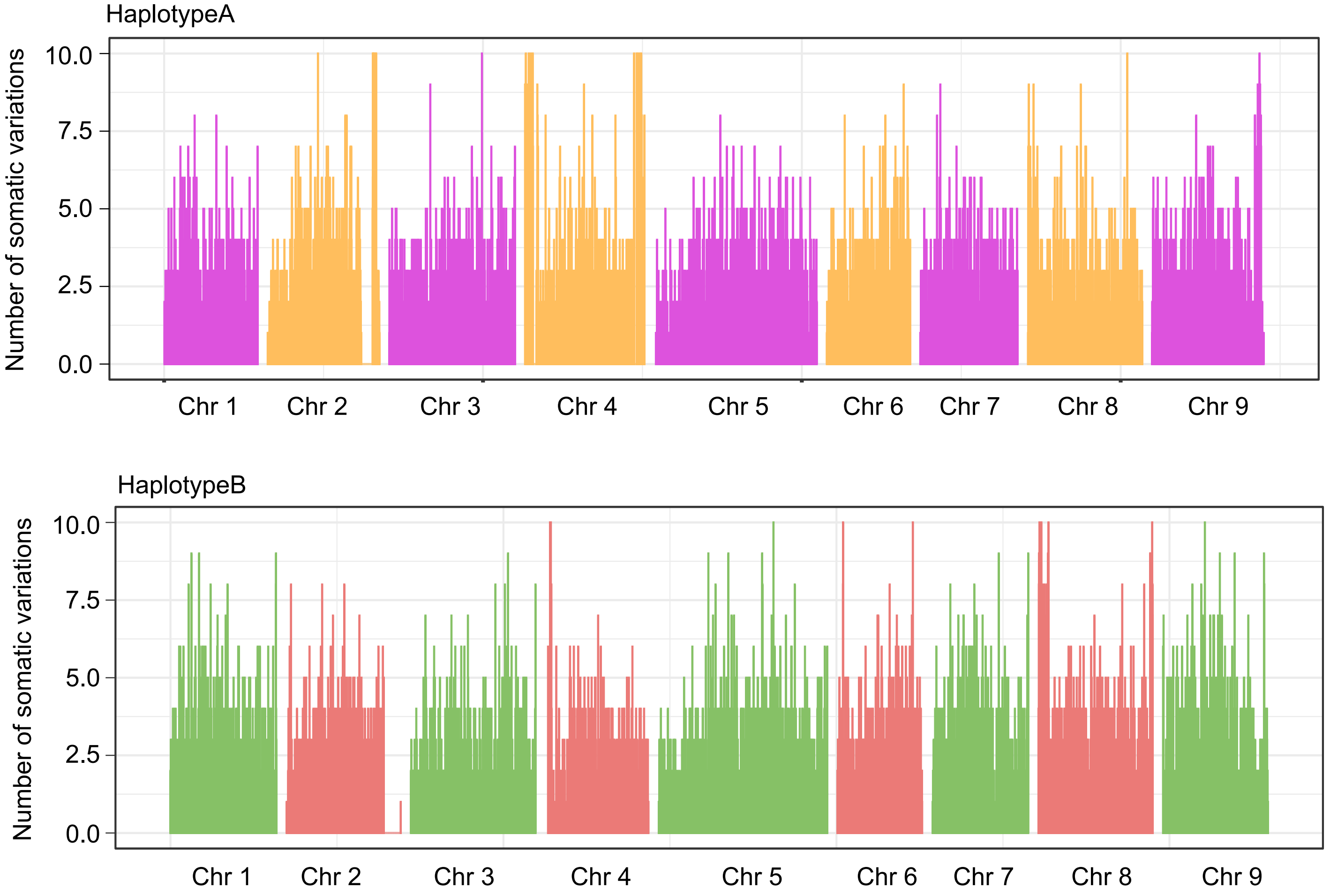

Supplementary Fig. 14. Distribution of somatic mutations in non-overlapping 50-kb windows in haplotypes A and B. The y-axis indicates the number of somatic mutations in each window.

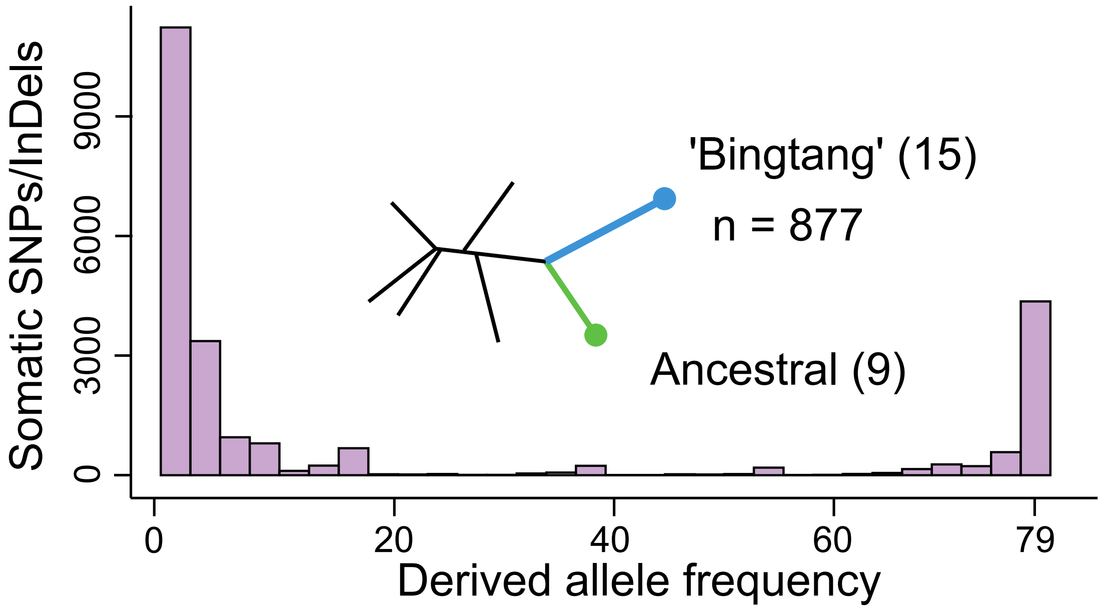

Supplementary Fig. 15. Somatic frequencies in 78 accessions. The ancestral state was inferred based on nine accessions from an Earlier Clade I. The numbers of Bingtang lineage (15 samples) specific somatic mutations (n=877) are highlighted.

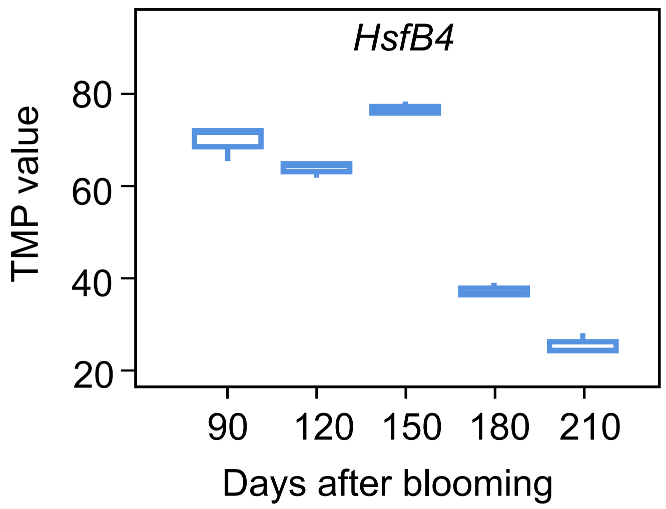

Supplementary Fig. 16. Expression of the *HsfB4* gene during five different developmental stages (90, 120, 150, 180 and 210 d after blooming) of fruit development. The TPM values are indicated on the y-axis.

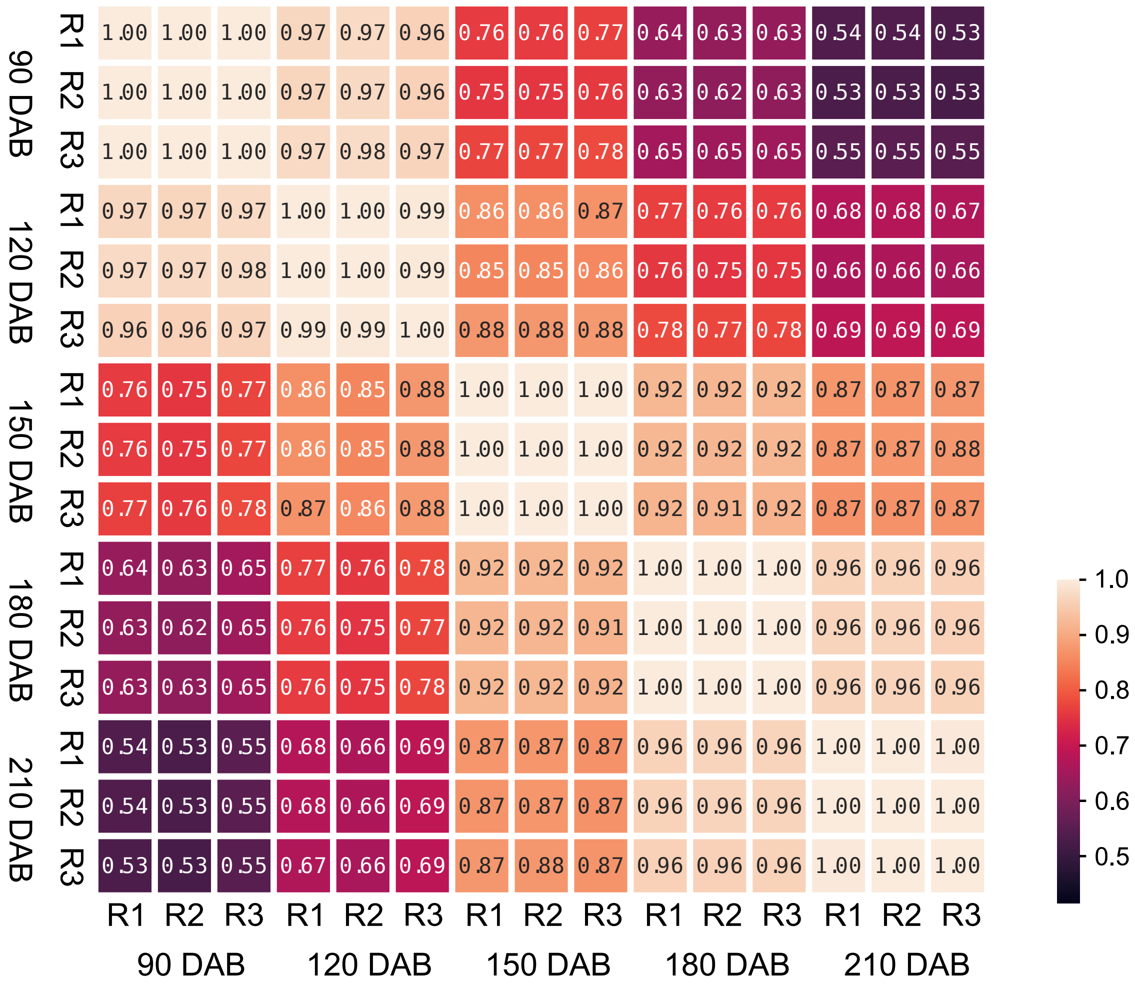

Supplementary Fig. 17. Correlations between sweet orange fruit and RNA-seq data at 90, 120, 150, 180 and 210 d after blooming (DAB). Three biological repeats are labeled R1, R2 and R3.

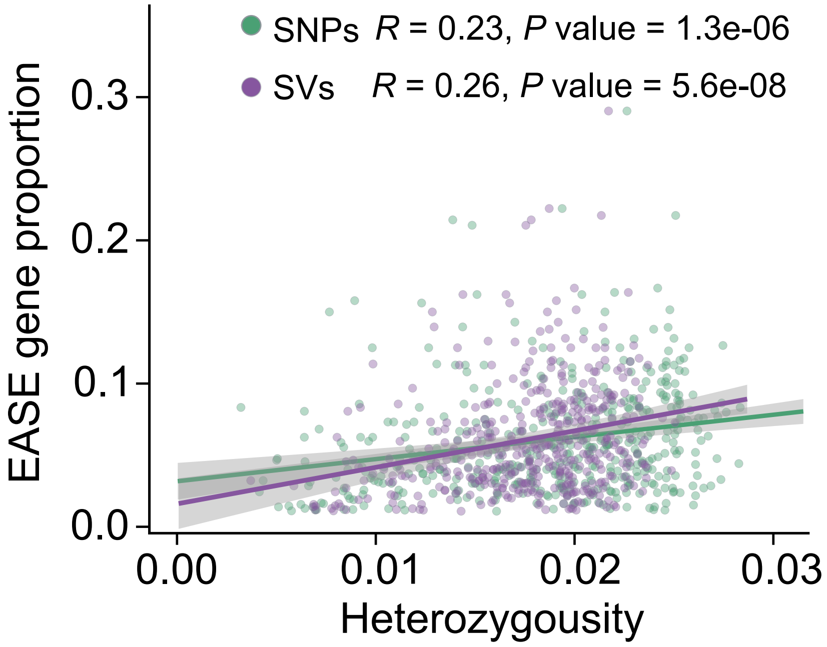

Supplementary Fig. 18. Correlation analysis between EASE genes and heterozygous SVs. The proportion was calculated based on the number of EASE genes and the number of genes in each 500-kb window.

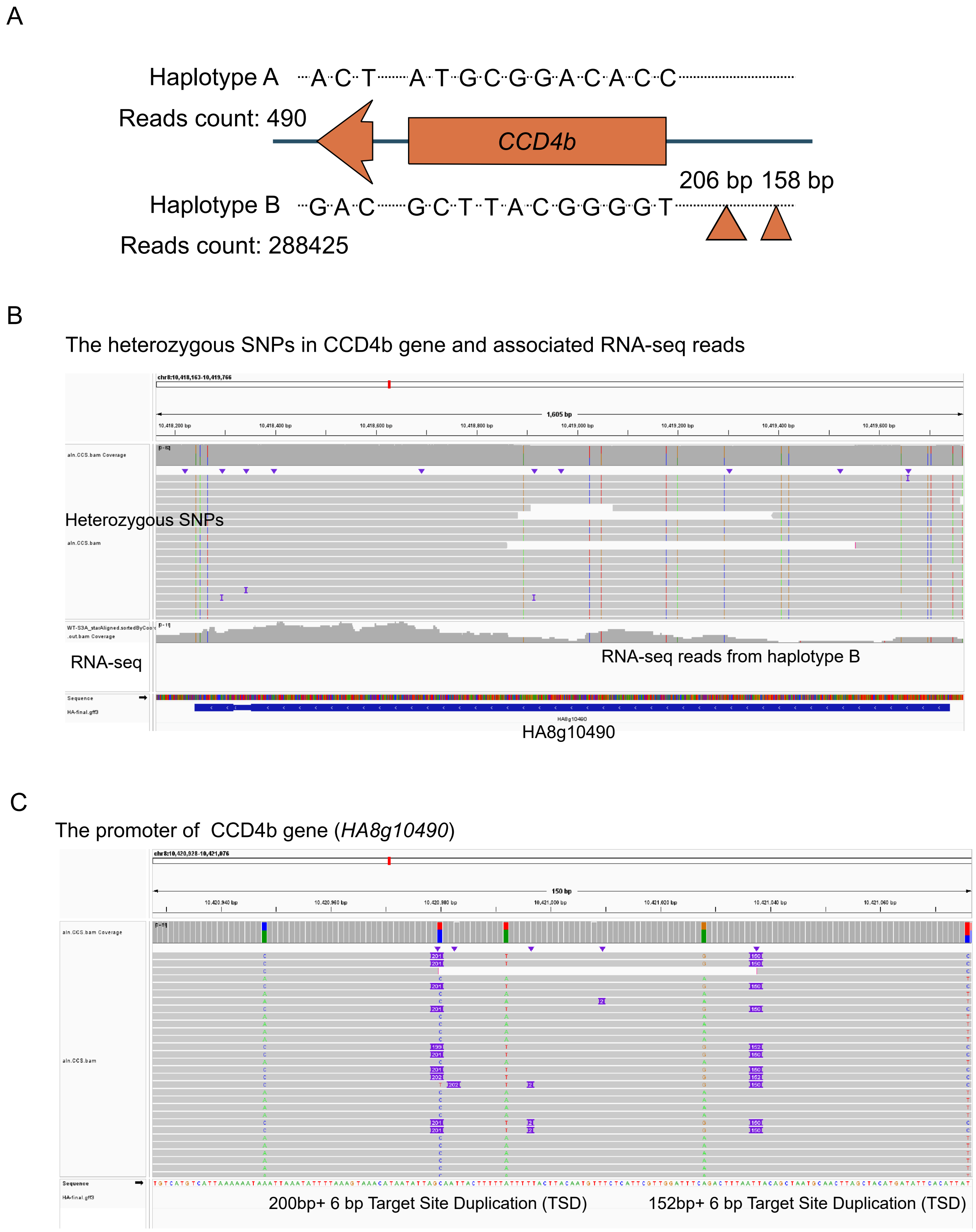

Supplementary Fig. 19. Heterozygous variations in the *CCD4b* gene body and promoter. (A) Heterozygous SNPs in the gene body of *CCD4b* and two heterozygous MITE insertions (206 bp and 158 bp) in the promoter of *CCD4b*. The counts from allele-specific reads are shown. (B) IGV plot of heterozygous SNPs in the *CCD4b* gene body based on HiFi reads and RNA-seq reads. (C) IGV plot of two heterozygous MITE insertions (206 bp and 158 bp) in the promoter of the *CCD4b* gene based on HiFi reads.

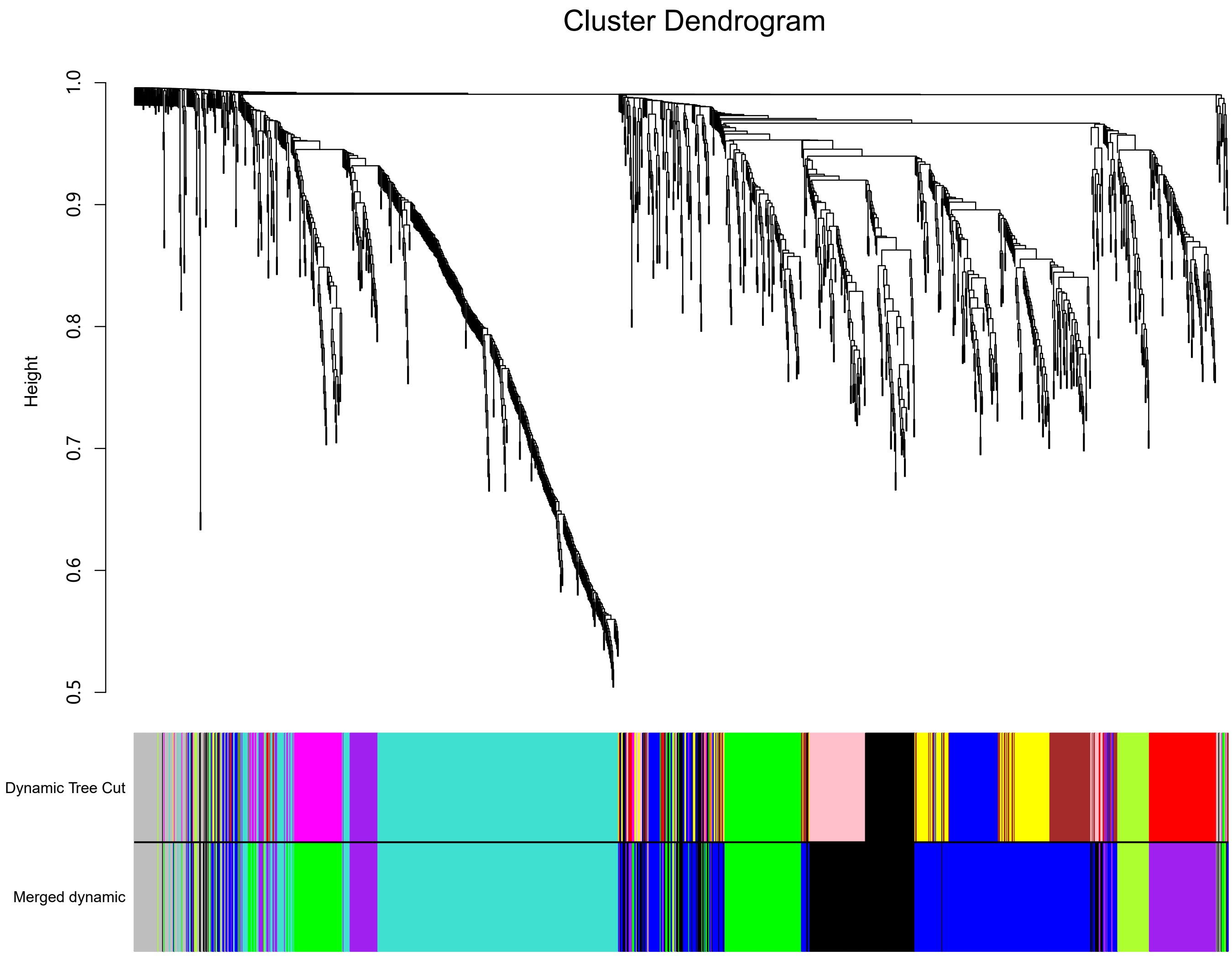

Supplementary Fig. 20. Clustering dendrograms and potential modules from the WGCNA. The cluster analysis was performed based on the expression of 3,172 extreme allelic differentially expression (EASE) genes.

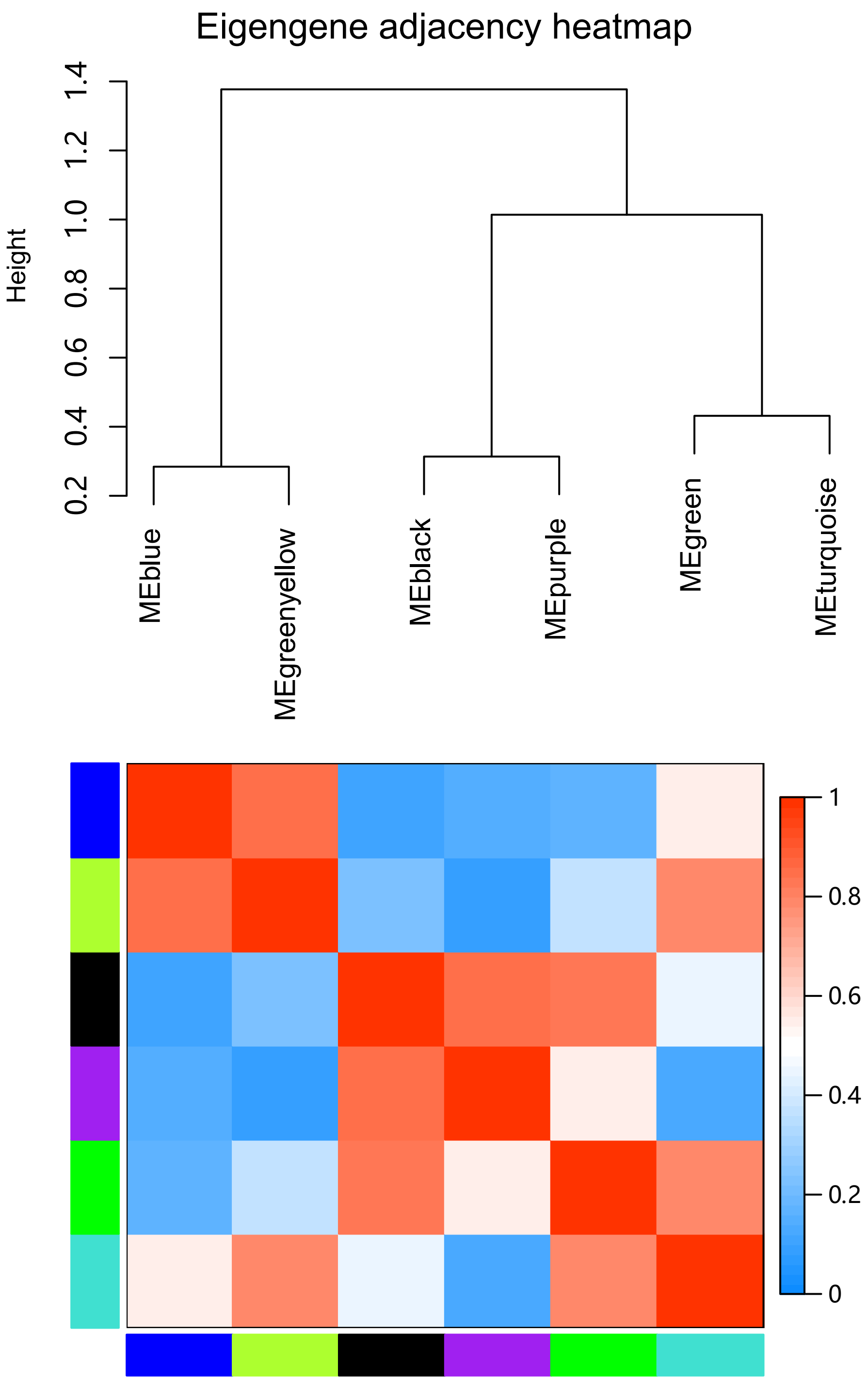

Supplementary Fig. 21. Correlations among seven major modules from the WGCNA based on the eigengene adjacency heatmap.

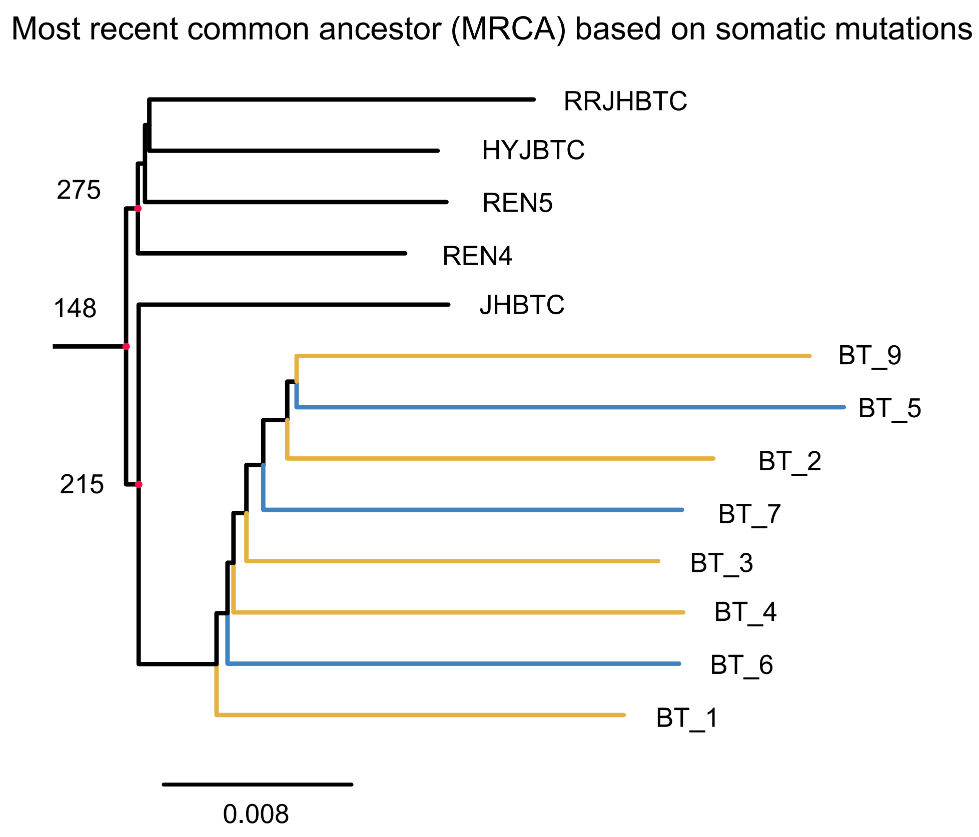

Supplementary Fig. 22. Clusters of sweet oranges that produce large fruit or wild-type fruit based on somatic mutations. Three newly sequenced samples that produce large fruit are highlighted in blue (BT_5, BT_6 and BT_7). The remaining five newly sequenced samples are highlighted in yellow. The number of conserved somatic mutations is marked in nodes.

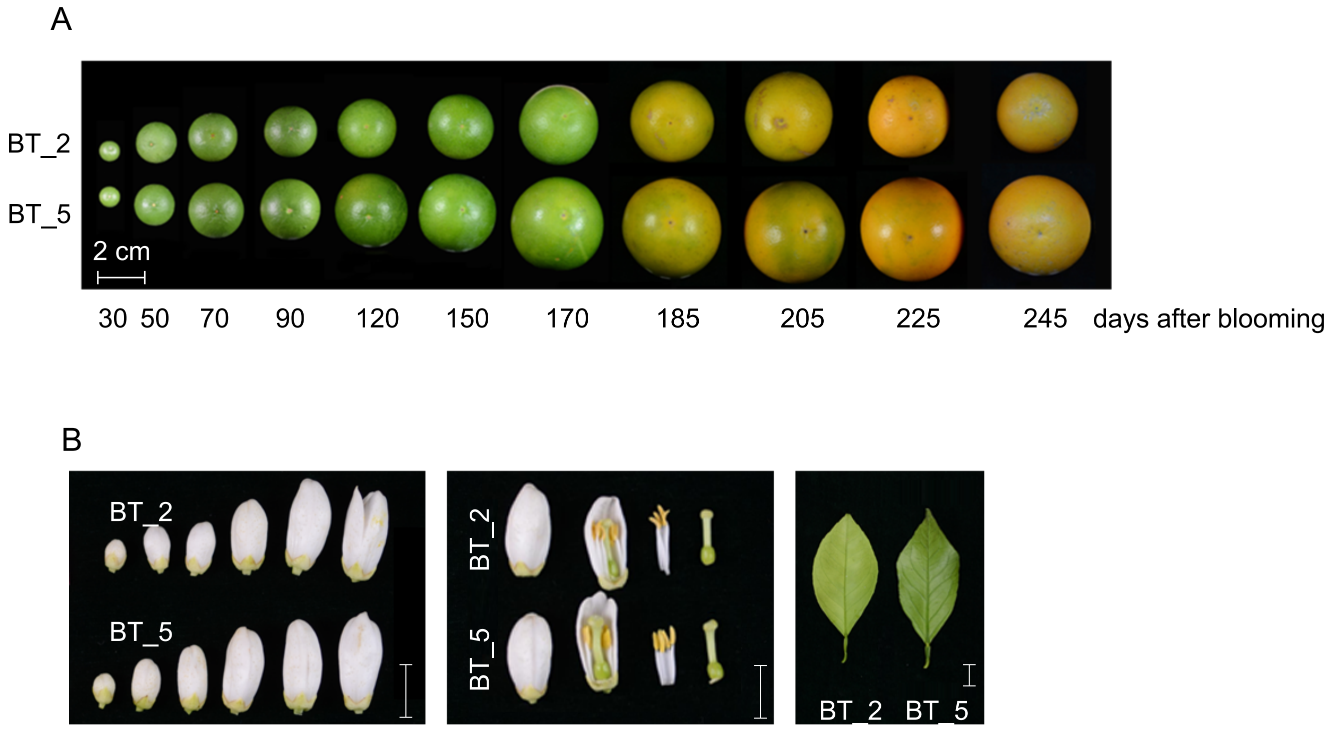

Supplementary Fig. 23. Morphological analysis of BT_2 and BT_5 sweet orange. (A) Fruit development in BT_2 and BT_5 at different stages. Scale bar, 2 cm. (B) Flowers and leaves from BT_2 and BT_5. Scale bar, 2cm.

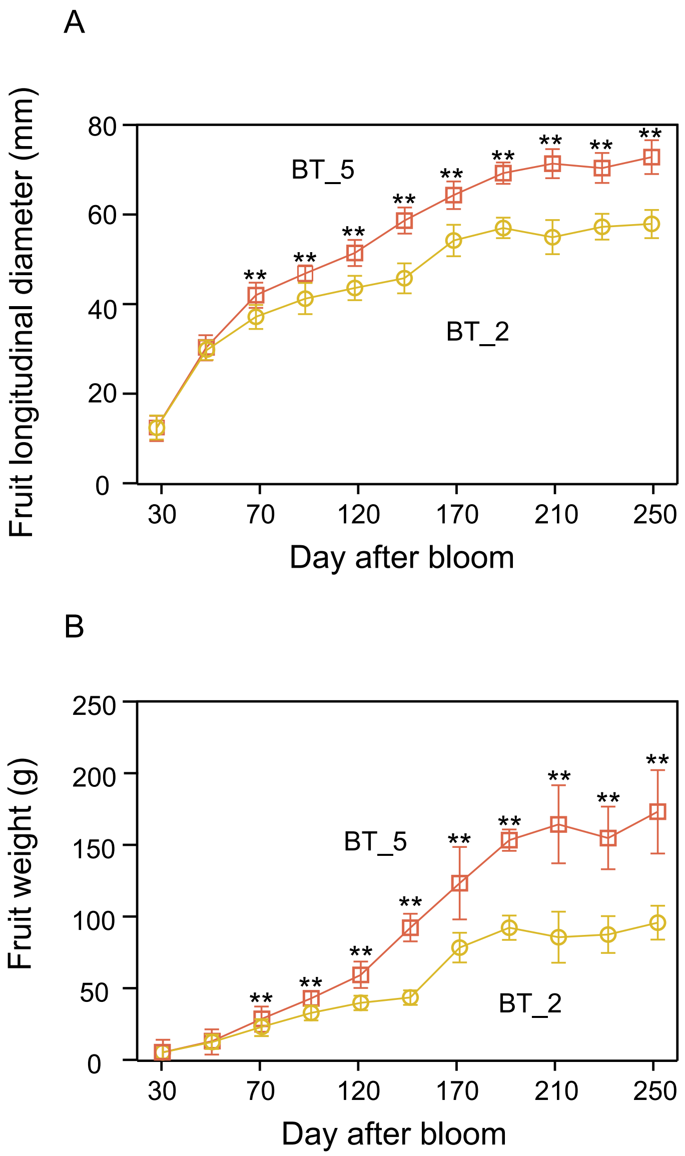

Supplementary Fig. 24. Changes in fruit longitudinal diameter (mm) and fruit weight (g) during 11 different developmental stages. * P value < 0.05; ** P value < 0.01 (Student t-test).

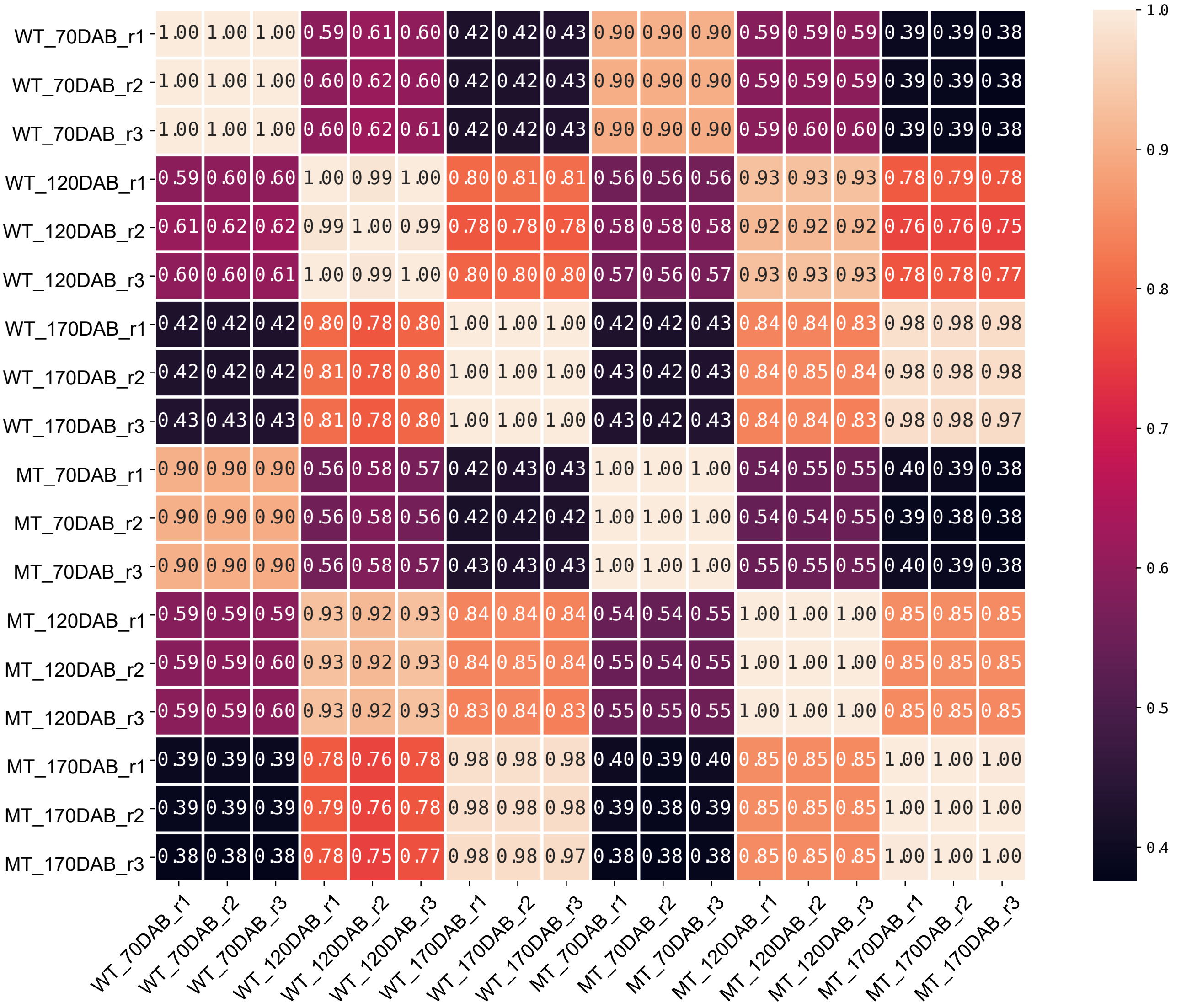

Supplementary Fig. 25. Correlations between sweet orange fruit RNA-seq data from BT_2 (WT, normal fruit size) and BT_5 (MT, large-fruit size) at 70, 120 and 170 d after blooming (DAB). Three biological replicates are labeled r1, r2 and r3.

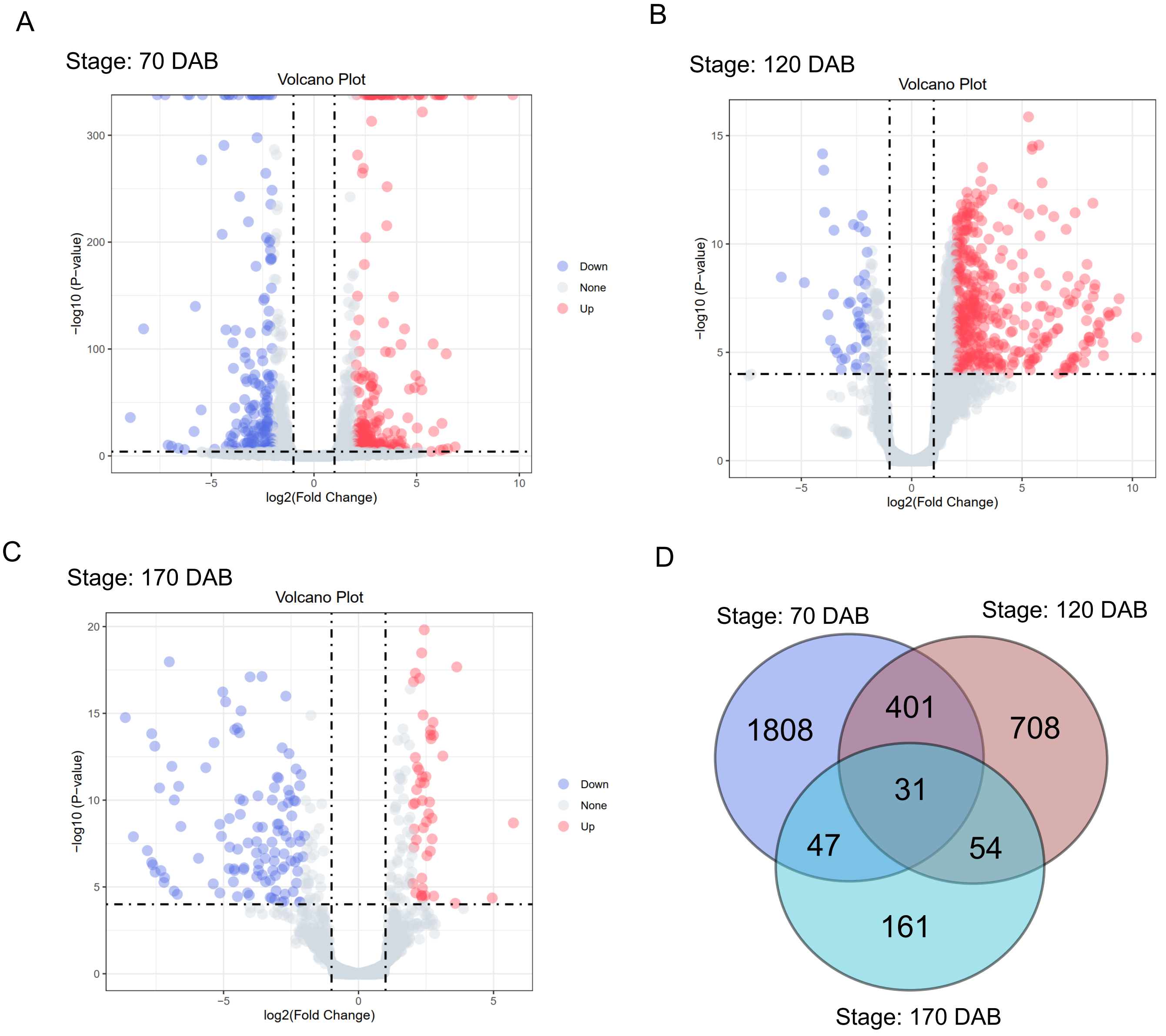

Supplementary Fig. 26. Genes that are differentially expressed in BT_2 relative to BT_5 at three different stages of development. (A-C) Volcano plot of differentially expressed genes (DEGs). The downregulated DEGs and upregulated DEGs are highlighted with different colors. (D) Venn diagram of DEGs from three different developmental stages.

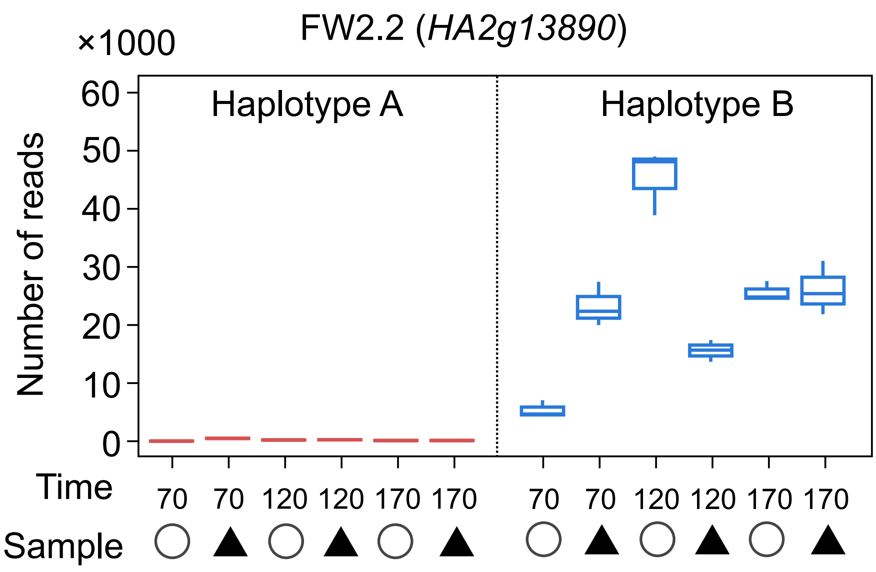

Supplementary Fig. 27. Expression of *FW2.2* (HA2g13890) gene alleles in BT_2 relative to BT_5 during three different stages of fruit development. The number of RNA-seq reads from the alleles in haplotypes A and B are indicated on the y-axis.
